# Just-in-Time Adaptive Intervention for Smoking Cessation in Low-Income Adults: A Randomized Clinical Trial

**DOI:** 10.1001/jamanetworkopen.2025.26691

**Published:** 2025-08-14

**Authors:** Emily T. Hébert, Darla E. Kendzor, Damon J. Vidrine, Jeremy S. Langford, Krista M. Kezbers, Audrey Montgomery, Meng Chen, Summer G. Frank-Pearce, Sara K. Vesely, Sixia Chen, Zachary C. W. Barrett, Michael S. Businelle

**Affiliations:** 1 TSET Health Promotion Research Center, Stephenson Cancer Center, University of Oklahoma Health Sciences, Oklahoma City; 2 Department of Family and Preventive Medicine, University of Oklahoma Health Sciences, Oklahoma City; 3 Department of Health Outcomes and Behavior, Moffitt Cancer Center, Tampa, Florida; 4 Department of Oncologic Sciences, Morsani College of Medicine, University of South Florida, Tampa; 5 Department of Psychology, University of Southern California, Los Angeles; 6 Department of Biostatistics and Epidemiology, Hudson College of Public Health, University of Oklahoma Health Sciences, Oklahoma City

## Abstract

**Importance:**

Smoking prevalence remains alarmingly high among low-income adults, who face disproportionate barriers to cessation and experience unique smoking lapse triggers.

**Objective:**

To compare the efficacy of the Smart-T smartphone intervention, featuring tailored, just-in-time adaptive interventions, with the National Cancer Institute QuitGuide intervention in supporting smoking cessation among adults with low income.

**Design, Setting, and Participants:**

This randomized clinical trial was conducted from August 2019 to November 2023 among US adults with household incomes below 200% of the federal poverty line. Participants were randomized to Smart-T or QuitGuide and followed-up for 27 weeks. Data were analyzed from July 2024 to March 2025.

**Interventions:**

Both groups received nicotine replacement therapy. The Smart-T intervention provided ecological momentary assessment–driven tailored feedback and prompts to use nicotine replacement therapy, while QuitGuide delivered smoking cessation content based on clinical guidelines.

**Main Outcomes and Measures:**

The primary outcome was biochemically verified 7-day point prevalence abstinence at 26 weeks after quitting, and group differences were evaluated via multivariable logistic regression. Secondary outcomes included 30-day point prevalence abstinence and continuous abstinence. Outcomes were analyzed using logistic regression with intention-to-treat and complete-case approaches.

**Results:**

A total of 454 participants (mean [SD] age, 52.0 [11.2] years; 333 [73.3%] female; mean [SD], 17.7 [9.5] cigarettes/d) were enrolled, with 225 randomized to Smart-T and 229 randomized to QuitGuide. Biochemically verified 7-day point prevalence abstinence at 26 weeks was higher in the Smart-T group than in the QuitGuide group (37 participants [16.4%] vs 23 participants [10.0%] in intention-to-treat analysis; 37 of 160 participants [23.1%] vs 23 of 161 participants [14.3%] in complete-case analysis). Adjusted odds of abstinence were significantly higher for Smart-T (adjusted odds ratio, 1.81; 95% CI, 1.03-3.18). No significant differences were found for 30-day or continuous abstinence. Smart-T users interacted with the app more often and rated it as more helpful (*r* = 0.21; *P* < .001).

**Conclusions and Relevance:**

In this randomized clinical trial of 454 adults with low income, the Smart-T intervention led to significantly higher smoking abstinence at 26 weeks compared with QuitGuide. These findings suggest that digital health interventions tailored for low-income populations hold promise for reducing smoking-related health disparities.

**Trial Registration:**

ClinicalTrials.gov Identifier: NCT03740490

## Key Points

**Question:**

Does a just-in-time adaptive intervention app that messages based on real-time lapse risk improve smoking cessation compared with the National Cancer Institute QuitGuide app among low-income adults?

**Findings:**

In this randomized clinical trial of 454 adults with low income, a higher percentage of users of the adaptive app achieved biochemically verified smoking abstinence at 26 weeks compared with QuitGuide users. Adaptive app users also used the app more frequently and rated it as more helpful.

**Meaning:**

These findings suggest that personalized, real-time digital interventions may improve smoking cessation among low-income populations, supporting broader adoption of adaptive mobile health tools.

## Teaser

This randomized clinical trial compares the efficacy of the Smart-T smartphone intervention, featuring tailored, just-in-time adaptive interventions, with the National Cancer Institute QuitGuide app in supporting smoking cessation among adults with low income.

## Introduction

Tobacco smoking remains the leading preventable cause of death in the United States, reducing life expectancy by a mean of 12 years.^1,2,3^ While smoking rates in the US have declined,^4^ prevalence remains high among low-income adults, contributing to persistent health disparities.^5^ Traditional smoking cessation programs are often less effective for individuals with lower socioeconomic status (SES), despite comparable motivation and attempts to quit.^6,7,8^ Addressing these disparities requires interventions that specifically target cessation barriers^9^ and smoking lapse triggers, including exposure to others who smoke, nicotine cravings, and stress.^7,8^

The growing adoption of smartphones among individuals with low SES provides an opportunity to deliver timely and tailored cessation support.^10,11^ Digital interventions, including smartphone apps, have been shown to increase cessation rates compared with minimal or no support.^12,13^ However, many apps do not follow evidence-based guidelines,^14,15^ and few randomized clinical trials (RCTs) have evaluated their efficacy using biochemically verified outcomes.^16,17,18^ Studies^19^ report promising engagement and cessation outcomes but findings are often limited by small sample sizes and lack of appropriate control groups. A recent fully powered RCT comparing an app-based intervention with brief advice demonstrated significantly higher abstinence rates in the intervention group,^20^ reinforcing the potential of mobile health interventions. Notably, apps combined with pharmacotherapy improve cessation success, while standalone apps without pharmacological support often fail to outperform control conditions.^16^ Personalized or interactive features and adherence to behavioral theories may enhance the effectiveness of digital interventions.^21,22^

Ecological momentary assessment (EMA),^23^ a mobile real-time data collection method, has emerged as a valuable tool for identifying and addressing the immediate determinants of smoking lapses.^24,25,26^ EMA can detect high-risk moments by capturing dynamic changes in psychological, social, and environmental factors. For example, EMA data have been used to identify increased lapse risk within 4 hours of a smoking event, driven by factors such as heightened urges, stress, alcohol use, and interaction with others who smoke, as well as reduced cessation motivation.^27,28^ Just-in-time adaptive interventions (JITAIs),^29,30^ which deliver adaptive, real-time support based on ecological momentary assessment (EMA) data,^31^ show promise in improving cessation outcomes.^11,27,28^ These interventions adjust dynamically to lapse triggers, potentially enhancing their reach and effectiveness for adults with low SES,^22^ who often face barriers to cessation programs.^9^ The Smart-T intervention was designed to overcome these challenges by delivering automated, tailored support directly through participants’ smartphones.^28,32^

This study is the first fully powered RCT to compare the Smart-T intervention with another smartphone-based smoking cessation intervention using biochemically verified abstinence as the primary outcome. We evaluated the efficacy of the Smart-T intervention^28,32^ compared with the National Cancer Institute (NCI) QuitGuide app,^33^ both combined with nicotine replacement therapy (NRT), over 26 weeks after a scheduled quit attempt.

## Methods

**Figure 1.**
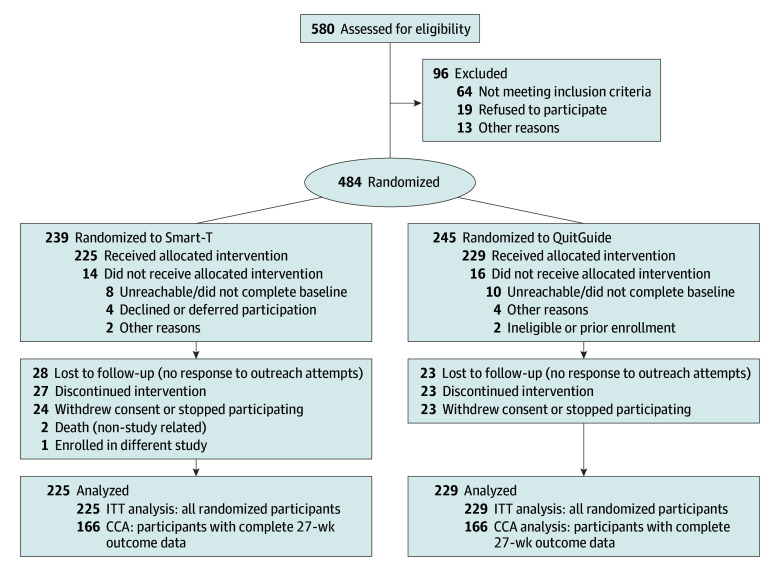
Participant Enrollment Flowchart CCA indicates complete case analysis; ITT, intention-to-treat.

This RCT was approved by the institutional review board at the University of Oklahoma Health Sciences (OUHS). All participants provided written informed consent. The trial was completed as planned, with no early termination. The full study protocol is available in Supplement 1. This report follows the Consolidated Standards of Reporting Trials (CONSORT) reporting guideline for RCTs (Figure 1).

### Participants and Procedure

Participants were recruited through the TSET Health Promotion Research Center Tobacco Treatment Research Program and through nationwide advertisements (eg, social media advertisements). The Tobacco Treatment Research Program provides free smoking cessation services and facilitated the recruitment, screening, and enrollment of participants. Recruitment through advertisements was managed separately by the study team.

Eligibility criteria included: (1) a score of at least 4 on the Rapid Estimate of Adult Literacy in Medicine–Short Form, a validated measure assessing health-related reading ability through recognition of common medical words (indicating literacy above the 6th-grade level); (2) willingness to quit smoking within 7 days of baseline; (3) age at least 18 years; (4) exhaled carbon monoxide (co) level at least 7 ppm (in person or remotely verified); (5) smoking at least 5 cigarettes/d; (6) no contraindications for NRT, including uncontrolled blood pressure, recent myocardial infarction (within 2 weeks), or current or planned pregnancy; (7) agreement to complete EMAs and co tests using a smartphone and Bedfont iCO Smokerlyzer for 27 weeks; (8) household income 200% or less of the federal poverty guideline (aligned with federal low-income definitions, eg, Medicaid); and (9) agreement to complete the 26-week follow-up. Exclusion criteria included noncitizens, those unable to verify residency or Social Security number, or employment at the University of Oklahoma. Data collection occurred from August 2019 to November 2023, with each participant followed-up for 27 weeks, including a final assessment at 26 weeks after the quit date (Figure 1).

### Study Design

This 2-group parallel RCT assigned eligible participants (1:1) to either the Smart-T smoking cessation intervention plus NRT or the NCI QuitGuide smoking cessation intervention plus NRT. Randomization was conducted using a blocked, stratified approach, balancing sex (male or female), race (White or not White, including Black participants and those who identified as other race [eg, American Indian/Alaska Native, Asian, and individuals who selected >1 race]), and cigarettes per day (CPD; <15 or ≥15), with a block size of 6. The allocation sequence was generated by the study statistician using the REDCap (Vanderbilt) automated randomization module, which concealed assignment until the moment of randomization. A slight imbalance in group sizes resulted from participants who were deemed ineligible after randomization and were excluded from analyses. Study staff enrolled participants and assigned interventions through the REDCap system at the time of baseline assessment. Following baseline, participants received instructions on completing EMAs, co assessments, and follow-up assessments via smartphone. Due to the COVID-19 pandemic, the protocol was amended to allow remote baseline visits via phone and mail, incorporate COVID-19 vaccine and pandemic-related stress assessments, implement additional participant contact methods (eg, delinquency letters, social media outreach), and expand recruitment nationwide. All participants received a 4-week supply of free NRT at baseline. Participants could request additional NRT throughout the study at no cost.

### Smart-T Intervention

Participants assigned to Smart-T downloaded the Insight app, which housed all surveys and intervention content. The 27-week Smart-T intervention included automated and on-demand content. Automated messages, delivered immediately at the end of each EMA, were tailored based on the participant’s name and momentary risk of smoking lapse.

The app included more than 400 expert-reviewed messages^34^ categorized into 4 levels: (1) level 0 messages (before the quit date) focused on motivation and quit planning; (2) level 1 messages (after the quit date, low-risk)^27^ reinforced abstinence and provided general cessation advice; (3) level 2 messages (high-risk moments, eg, recent smoking or high likelihood of smoking) offered tailored coping strategies; and (4) level 3 messages (after a lapse) encouraged a return to abstinence. When lapse risk was high,^27^ participants were prompted with “Chewing a piece of nicotine gum or using a nicotine lozenge right now may reduce your risk for smoking. Will you chew a piece of nicotine gum or use a nicotine lozenge right now?”

**Figure 2.**
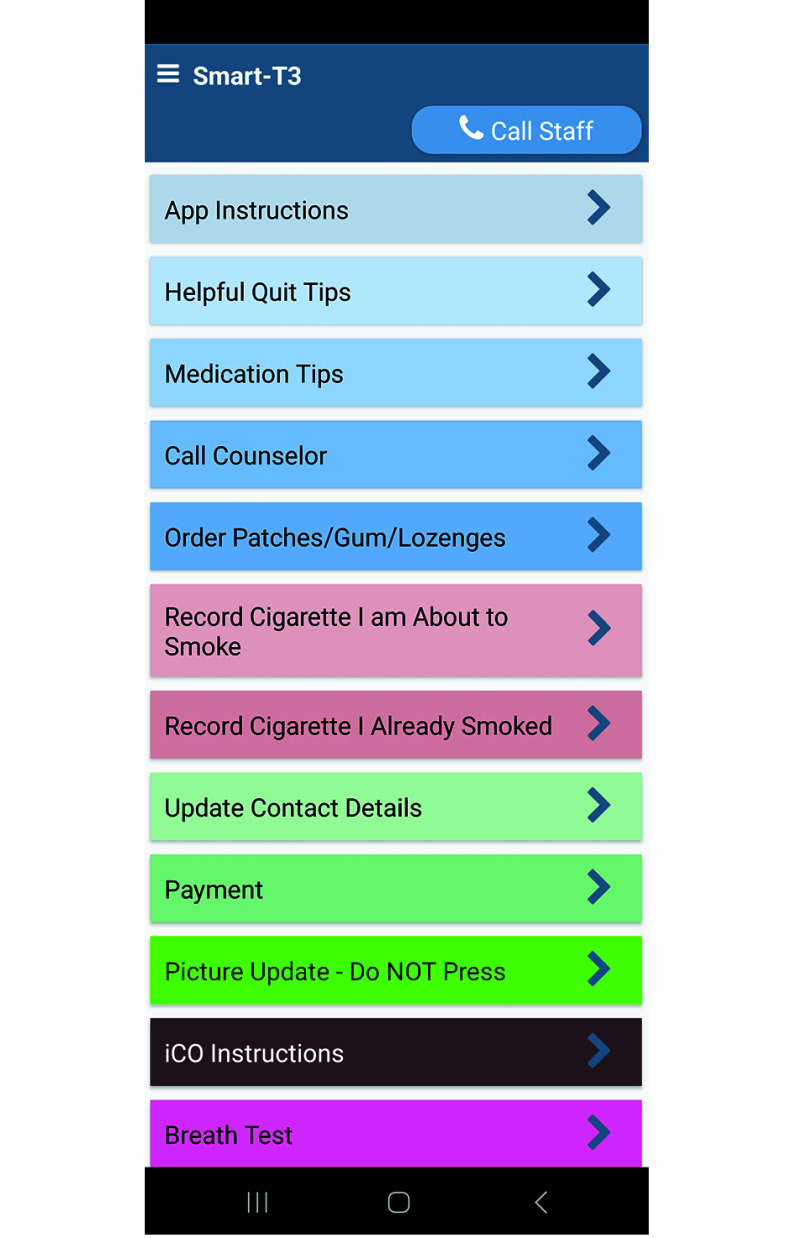
Screenshot of the Smart-T App This figure illustrates the main interface of the Smart-T app, which provides participants with access to key smoking cessation resources. Features include app instructions, helpful quit tips, medication tips, a call counselor button, options to order nicotine replacement therapy (patches, gum, lozenges), a tool to record cigarette use, payment tracking, contact update functionality, and a direct call option for study staff support.

On-demand features included a “Call Counselor” button for immediate quitline support, a “Quit Tips” button with treatment-related messages, and a “Medications” button with NRT information (Figure 2).^28,32^ Participants also received daily reminders of their scheduled quit date during the prequit period.

### NCI QuitGuide Intervention

Participants in the QuitGuide group downloaded the app onto personal or study-provided smartphones. The app, based on clinical practice guidelines,^35^ offered smoking cessation support, including tracking cravings, mood, smoking triggers, and quit motivations. It also provided information on cessation medications, coping strategies, and a personalized quit plan. Participants received enrollment instructions and were encouraged to use the app throughout the 27-week study. Additionally, they downloaded the Insight app for completing EMAs.

### Compensation

Participants received $30 for the baseline assessment and up to $55 for the 26-week follow-up, including a $5 bonus for timely completion. EMA incentives were based on biweekly completion rates, with up to $449 available over the 27-week study. Payments were delivered via Greenphire gift cards. During weeks with higher EMA frequency (up to 5 prompts per day), participants could earn up to $50 per 15-day period. In later weeks (1-3 prompts per day), the maximum ranged from $30 to $36 per period. Payments were prorated based on completion, with full compensation awarded for at least 90% of prompts completed. Participants were not compensated for accessing app features or completing participant-initiated assessments.

### Measures

#### Baseline and Follow-Up

Baseline assessments collected demographic information, smoking history (eg, heaviness of smoking index, mean number of cigarettes smoked per day), and psychosocial constructs related to smoking lapse, including depression,^36^ stress,^37^ affect,^38^ and social support.^39^ Race and ethnicity were self-reported by participants via the baseline survey. Race and ethnicity were assessed to examine potential differences in smoking cessation outcomes and to control for sociodemographic factors that may influence treatment response. The 26-week postquit follow-up assessment, which included baseline measures and treatment perception and engagement questions,^32^ was completed via the Insight app.

#### EMA

All participants, regardless of treatment group, completed EMAs using the Insight app. EMA was used to assess real-time behaviors in natural settings through daily diary, random sampling, and event sampling. Daily diary EMAs were completed twice daily during weeks 1 to 14, with a morning diary prompted 30 minutes after the participant’s scheduled wake time and an evening diary prompted 60 minutes before their scheduled bedtime. From weeks 15 to 27, participants completed a single end-of-day diary only. Random sampling EMAs were prompted 3 times daily at semirandom times within the participant’s self-reported waking hours for the first 5 weeks and once daily for weeks 6 through 14. Event sampling EMAs were self-initiated by participants to record smoking (precessation) or lapse (postcessation) events. Each EMA lasted approximately 2 to 3 minutes.

EMA items included recent smoking, stress, smoking urges, cigarette availability, cessation motivation, alcohol use, and social interactions.^27,28^ Daily diary EMAs also included breath co tests 3 days per week throughout the 27-week study (Sundays, Tuesdays, and Thursdays). Facial recognition software was integrated into the study app to verify participant identity during co testing. Use of the iCO Smokerlyzer and Insight mHealth app ensured consistent biochemical verification procedures across treatment groups.

### Smoking Cessation Outcomes

The primary outcome was 7-day point prevalence abstinence (PPA) at 26 weeks after the quit date, defined as self-reported abstinence in the past 7 days with co 6 ppm or less.^40^ Secondary outcomes included 30-day PPA at 26 weeks, defined as self-reported abstinence in the past 30 days with co 6 ppm or less, and continuous abstinence, defined as abstinence since the quit date with co 6 ppm or less at the follow-up. Missing data were handled via complete case analysis (CCA) and intent-to-treat (ITT) analysis, with ITT assuming participants lost to follow-up had relapsed.

### Participant Perceptions and Engagement

At the 27-week follow-up, participants completed a brief self-report survey assessing their perceptions of the intervention app and their experience with smartphone-based assessments. Items covered perceived burden (eg, “Did the smartphone assessments ever make you want to smoke?”), self-awareness (eg, “Did carrying the phone and answering questions make you more aware of your thoughts, feelings, and behavior?”), perceived helpfulness, satisfaction with treatment, and likelihood of recommending or reusing the app. Responses were rated on Likert-type scales with varying ranges depending on the item (eg, 0 to 5, 1 to 7), with higher score indicating the more favorable outcome.

Engagement with the intervention apps was measured using backend app metadata that captured participants’ use of specific app features, including the number of times on-demand messages were accessed, automated messages were viewed, and various in-app tools were used (eg, mood tracking). Engagement data were summarized separately for each intervention group.

### Data Management

Data were collected and managed using the Insight mHealth Platform,^41^ REDCap, and Questionnaire Development System. The Insight mHealth Platform, developed by the mHealth Shared Resource at OUHS and Stephenson Cancer Center,^42^ enabled secure data collection and storage. Data were password-protected and encrypted on smartphones and transmitted to a secure server. Baseline assessments were conducted in person using the Questionnaire Development System or remotely using REDCap, while follow-up survey data were collected via Insight or REDCap.

### Statistical Analysis

Demographic and baseline smoking characteristics were summarized using means and SDs for continuous variables, and frequencies and percentages for categorical variables. Age, cigarettes smoked per day, and the Heaviness of Smoking Index were treated as continuous variables. Sex, race, ethnicity, education, and income were treated as categorical. Educational attainment was dichotomized as less than high school vs high school or greater. Annual household income was dichotomized as less than $30 000 vs $30 000 or more to align with federal poverty thresholds and study eligibility criteria. Group comparisons used χ^2^ tests, *t* tests, or nonparametric tests (ie, Mann-Whitney *U* tests) as appropriate. Logistic regression analyses evaluated 7-day, 30-day, and continuous abstinence at 26 weeks after the quit date, with treatment group (0 = QuitGuide, 1 = Smart-T) as the primary predictor. Unadjusted odds ratios (ORs) and adjusted ORs (AORs) were calculated, controlling for age, race (categorized as White or not White), sex, and baseline cigarette consumption. The primary analysis was conducted using an ITT approach, in which participants lost to follow-up were assumed to be smoking. A CCA was also conducted as a sensitivity analysis. All analyses were conducted in SPSS software version 26 (IBM). All statistical tests were 2-sided, and a significance level of *P* < .05 was used. The sample size calculation (N = 450; 225 per group) was based on the following assumptions: (1) 7-day PPA rates of 18% in Smart-T vs 9% in QuitGuide at 26 weeks, (2) equal allocation between groups, (3) a 2-sided type I error rate of 0.05, and (4) 80% power to detect a 9% absolute difference. A dropout rate was not explicitly incorporated, as missing data were handled using ITT and CCA. Data were analyzed from [date] to [date].

## Results

### Participants

A total of 454 participants (mean [SD] age, 52.0 [11.2] years; 333 [73.3%] female; mean [SD], 17.7 [9.5] cigarettes/d) were enrolled, with 225 randomized to Smart-T and 229 randomized to QuitGuide. Participants were drawn from across the US, representing most states. By race and ethnicity, there were 111 Black participants (24.4%), 289 White participants (63.7%), and 54 participants (11.9%) who identified as other race; 23 participants (5.1%) were Hispanic. The only significant difference between the study groups was in the percentage of Hispanic participants (Table 1) (Cramér V = 0.092; *P* = .049), while all other demographic characteristics were similar. Most study participants completed their baseline visit remotely (326 participants [71.8%]), and 288 participants (63.4%) received a study-provided phone. Participant characteristics are provided in Table 1. Participant enrollment, including loss to follow-up and missing outcome data, is summarized in Figure 1. All 454 randomized participants were included in the ITT analyses. CCA samples included participants who completed the 27-week follow-up survey and provided outcome data, with sample sizes ranging from 321 to 332, depending on the specific abstinence outcome. There were no significant differences in follow-up rates across treatment groups (166 Smart-T participants [73.8%] vs 166 QuitGuide participants [72.5%]) (Figure 1).

**Table 1.** Participant Characteristics at Baseline

Characteristic	Participants, No. (%) (N = 454)		
	Total (n = 454)	Smart-T (n = 225)	QuitGuide (n = 229)
Age, mean (SD), y	52.0 (11.2)	52.0 (11.4)	52.0 (11.0)
Sex			
Female	333 (73.3)	166 (73.8)	167 (72.9)
Male	121 (26.7)	59 (26.2)	62 (27.1)
Race			
Black	111 (24.4)	52 (23.1)	59 (25.8)
White	289 (63.7)	143 (63.6)	146 (63.8)
Other^a^	54 (11.9)	30 (13.3)	24 (10.5)
Hispanic ethnicity	23 (5.1)	16 (7.1)	7 (3.1)
Educational level <high school	50 (11.0)	21 (9.3)	29 (12.7)
Annual household income <$30 000	323 (71.8)	157 (70.4)	166 (73.1)
Cigarettes smoked per day, mean (SD)	17.7 (9.5)	18.0 (10.3)	17.4 (8.6)
Heaviness of Smoking Index, mean (SD)	3.2 (1.4)	3.2 (1.4)	3.2 (1.4)

^a^ Includes American Indian or Alaska Native, Asian, and individuals who selected more than 1 race.

### Smoking Cessation Outcomes

Smoking cessation rates were higher in the Smart-T group compared with QuitGuide across all abstinence measures (Table 2). For 7-day PPA, the Smart-T group showed a higher abstinence rate compared with QuitGuide (37 participants [16.4%] vs 23 participants [10.0%] in ITT; 37 of 160 participants [23.1%] vs 23 of 161 participants [14.3%] in CCA). There were no statistically significant differences for 30-day PPA (33 participants [14.7%] vs 21 participants [9.2%] in ITT; 33 of 161 participants [20.5%] vs 21 of 163 participants [12.9%] in CCA) or continuous abstinence (25 participants [11.1%] vs 14 participants [6.1%] in ITT; 25 of 166 participants [15.1%] vs of 166 participants [8.4%] in CCA). Logistic regression analyses (Table 2) indicated that participants using Smart-T had significantly higher odds of 7-day PPA compared with those using QuitGuide (ITT: AOR, 1.81; 95% CI, 1.03-3.18; CCA: AOR, 1.85; 95% CI, 1.04-3.31), indicating a statistically significant benefit of Smart-T on abstinence at 26 weeks. The ORs for 30-day PPA (AOR, 1.75; 95% CI, 0.97-3.14) and continuous abstinence (AOR, 1.97; 95% CI, 0.99-3.92) did not reach statistical significance. Self-reported abstinence rates were substantially higher than biochemically verified rates across all outcomes (eTable 1 in Supplement 2). At 26 weeks, self-reported 7-day PPA included 129 participants (36.6%) overall, compared with 60 participants (18.7%) based on biochemical verification. Similar patterns were observed for 30-day abstinence (111 participants [32.0%] self-reported vs 54 participants [16.7%] verified) and continuous abstinence (69 participants [19.9%] self-reported vs 39 participants [11.7%] verified)

**Table 2.** Biochemically Verified Smoking Abstinence Rates and Associations With Intervention Group at 26 Weeks After Quit Date

Abstinence outcome	Participants, No./total No. (%)				(95% CI)	
	Total	Smart-T	QuitGuide	OR		aOR^a^
7 d						
ITT^b^	60/454 (13.2)	37/225 (16.4)	23/229 (10.0)	1.76 (1.01-3.08)^c^		1.81 (1.03-3.18)^c^
CCA	60/321 (18.7)	37/160 (23.1)	23/161 (14.3)	1.80 (1.02-3.21)^c^		1.85 (1.04-3.31)^c^
30 d						
ITT^b^	54/454 (11.9)	33/225 (14.7)	21/229 (9.2)	1.70 (0.95-3.04)		1.75 (0.97-3.14)
CCA	54/324 (16.7)	33/161 (20.5)	21/163 (12.9)	1.74 (0.96-3.17)		1.79 (0.98-3.27)
Continuous						
ITT^b^	39/454 (8.6)	25/225 (11.1)	14/229 (6.1)	1.92 (0.97-3.80)		1.97 (0.99-3.92)
CCA	39/332 (11.7)	25/166 (15.1)	14/166 (8.4)	1.93 (0.96-3.85)		1.94 (0.97-3.91)

Abbreviations: aOR, adjusted odds ratio; CCA, complete case analysis; ITT, intention-to-treat analysis; OR, odds ratio.

^a^ Adjusted for race (White or Black or other), age, sex, and baseline cigarettes per day.

^b^ In ITT analyses, participants with missing data were considered to be smoking.

^c^ Significant at *P* < .05.

### Treatment Engagement

#### Smart-T Use

Among 225 Smart-T participants, 209 (92.9%) accessed at least 1 on-demand “Helpful Quit Tip” item during the 26-week intervention. The most frequently accessed messages were “Ways to Cope with Urges” and “General Quitting Advice,” followed by medication-related content (eg, “Nicotine Patch Information,” 108 participants [51.7%] and “Nicotine Gum and Lozenges Information,” 103 participants [49.3%]). Less frequently accessed were messages about benefits of quitting, stress coping, and smoking harms (eTable 2 in Supplement 2). Among participants who used these features, the median number of messages viewed ranged from 10 to 18, with some participants viewing hundreds of messages within certain categories (eTable 3 in Supplement 2). A total of 183 participants (81.3%) used the “Call Counselor” feature, with a mean (SD) of 6.3 (6.4) uses per person (range, 1-36). Across participants, the app delivered 74 830 just-in-time treatment messages. Of these, 22 752 messages (30.4%) were low-risk messages (level 1). A total of 28 731 messages (38.4%) were level 2 messages triggered by high-risk lapse moments, addressing cigarette availability (7211 messages [25.1%]), smoking urges (1580 messages [5.5%]), stress or negative affect (1494 messages [5.2%]), and motivation to quit (747 messages [2.6%]). Additionally, 1777 messages (2.4%) were level 3 messages delivered immediately after a lapse, and 21 570 messages (28.8%) were level 4 messages encouraging abstinence on smoking days. In response to system-generated suggestions, participants agreed to use oral nicotine replacement therapy in 15 677 of 29 289 instances (53.5%).

#### QuitGuide Use

Data collected via the QuitGuide app could only be confirmed to match study identifiers for 156 participants (68.1%) randomized to the QuitGuide group. Among this subset, participants opened the app a median (range) of 3 (1-180) times throughout the 189-day study period. The most commonly used features included “I Was Smokefree Today” (80 participants [51.3%]; median [IQR], 6 [1-15] times; range, 1-194), “I Slipped” (95 participants [60.9%]; median [IQR], 3 [1-7] times; range, 1-72), and “How to Quit” (107 participants [68.6%]; median [IQR], 3 [1-6] times; range, 1-505).

Other features, such as “Manage My Mood,” “Track Craving,” and “Automated Tip Delivery,” were used by 32.7% to 62.2% of participants (eTable 4 in Supplement 2). Fewer participants used journaling (70 participants [44.9%]), quit date sharing (24 participants [15.4%]), or personalized statistics (81 participants [51.9%]). Use frequency varied widely across individuals and features.

### NRT

A total of 232 participants (51.1%) placed orders for additional free NRT during the study. Participants made a mean (SD) of 2.19 (1.23) NRT requests throughout the study, with the number of requests ranging from 1 to 6. More participants in the Smart-T group (140 participants [62.2%]) requested NRT compared with the QuitGuide group (92 participants [40.2%]). In addition, Smart-T participants requested NRT significantly more frequently (mean [SD], 2.51 [1.24] requests) than those in the QuitGuide group (mean [SD], 1.71 [1.05] requests) (*P* < .001; Cohen *d* = 0.69).

### Participant Perceptions of Their Intervention

At the end of the study, perceptions of the number of prompted EMAs and annoyance with the intervention did not differ significantly between groups (Table 3). However, compared with QuitGuide users, Smart-T users reported greater awareness of their thoughts and behaviors (*r* = 0.13; *P* = .02), rated the app as more helpful in supporting quitting efforts (*r* = 0.21; *P* < .001) and overall intervention helpfulness (*r* = 0.15; *P* = .01), and were more likely to recommend the app to others (*r* = 0.15; *P* = .005).

**Table 3.** Perceptions of Assigned App

Question	Respondents, No.	Mean (SD)	*P* value^a^
Did the smartphone assessments ever make you want to smoke?^b^			
QuitGuide	169	1.25 (1.32)	.18
Smart-T	164	1.07 (1.30)	
Did carrying the phone and answering questions make you more aware of your thoughts, feelings, and behavior?^c^			
QuitGuide	169	2.84 (0.89)	.02
Smart-T	164	3.07 (0.87)	
Did the assessments and messages help you to make decisions that were supportive of quitting and staying quit?^c^			
QuitGuide	169	2.78 (0.78)	<.001
Smart-T	163	3.08 (0.86)	
Do you find the smart phone application to be annoying?^d^			
QuitGuide	169	2.27 (1.08)	.21
Smart-T	163	2.23 (1.26)	
Overall, how helpful has the smartphone application been in helping you to quit smoking?^d^			
QuitGuide	169	2.96 (1.23)	<.001
Smart-T	163	3.46 (1.33)	
Overall, how helpful was the smoking cessation treatment that you received during this study?^d^			
QuitGuide	169	3.27 (1.26)	.007
Smart-T	163	3.63 (1.24)	
How likely would you be to recommend this smartphone app to a friend?^e^			
QuitGuide	169	5.32 (1.72)	.005
Smart-T	163	5.69 (1.78)	
Would you be interested in using this smartphone app in the future if needed?^f^			
QuitGuide	169	3.50 (1.31)	.08
Smart-T	163	3.71 (1.39)	

^a^ Group differences were evaluated using the Mann-Whitney *U* test due to the ordinal nature of Likert scale responses and potential nonnormality. Results are presented as means and SDs for descriptive purposes; however, statistical significance is based on nonparametric tests.

^b^ Responses ranged from never (0) to always (5).

^c^ Responses ranged from definitely no (1) to definitely yes (4).

^d^ Responses ranged from not at all (1) to extremely (5).

^e^ Responses ranged from extremely unlikely (1) to extremely likely (7).

^f^ Responses ranged from not at all interested (1) to extremely interested (5).

## Discussion

This RCT provides evidence that Smart-T, which tailored content in real time based on participant-reported lapse risk factors, outperformed the QuitGuide smoking cessation intervention. Smart-T demonstrated significantly higher biochemically verified 7-day PPA rates at 26 weeks, supporting the potential of mobile health interventions to reduce disparities in cessation outcomes among low-income adults.^5^

These findings build on prior work demonstrating the feasibility and acceptability of Smart-T.^28,32^ Participants frequently accessed messages targeting high-risk moments, reinforcing the app’s ability to address critical lapse triggers identified in EMA research.^25,27^

Smart-T’s real-time adaptive interventions align with prior evidence that personalized digital strategies outperform self-directed approaches.^21,22^ Prompts to use NRT during high-risk moments may have further enhanced cessation,^13^ but participants only accepted NRT suggestions in 53.5% of high risk for lapse moments, highlighting barriers, such as access or medication concerns. Future iterations of Smart-T could explore strategies to increase NRT use, such as addressing misconceptions, or integrating motivational content.

A key strength of this study is the inclusion of biochemically verified smoking abstinence as the primary outcome, reducing the risk of misclassification. This is particularly important in populations with high rates of misreporting due to demand characteristics or socioeconomic pressures.^43,44^ The discrepancy between self-reported and biochemically confirmed abstinence underscores the need for biochemical validation in future cessation research, particularly in high-risk populations.

### Limitations

This study has several limitations. Requiring participants to set a quit date exactly 7 days after enrollment may limit generalizability to individuals who are less ready to quit. Participants in the QuitGuide group used 2 separate apps (EMA and intervention), which may have reduced engagement, and database issues affected QuitGuide data completeness. Smart-T was available only on the Android operating system (Google) during the trial, although it has since been adapted for iOS (Apple), increasing scalability. Recruitment through digital platforms and a tobacco treatment program may limit generalizability to individuals with lower digital literacy or those not actively seeking cessation. While the 26-week follow-up captures end-of-treatment outcomes, future work should assess longer-term effects (eg, 6-12 months after treatment). Furthermore, this study was not designed to isolate the effects of individual intervention components (eg, EMA, JITAI, on-demand content). On-demand access to the quitline and other supports may have contributed to outcomes. Future studies using factorial or SMART designs are needed to identify which components drive effectiveness.

## Conclusions

These findings suggest that the Smart-T intervention shows promise as a low-cost, accessible tool for smoking cessation among adults with low SES and may help overcome barriers limiting traditional cessation programs. Future research should evaluate its effectiveness in a larger trial and explore additional components, such as stress management or social support. Given the ubiquity of smartphones, interventions like Smart-T could play a key role in reducing smoking-related health disparities and improving public health.

## Notes

Supplement 1.
Trial Protocol and Statistical Analysis Plan

Supplement 2. **eTable 1. **Self-Reported Smoking Abstinence Rates by Treatment Group Assignment at the 26-Week Postquit Follow-Up **eTable 2.** On-Demand Message Access Among Smart-T Participants **eTable 3.** Messages viewed per Category Among Smart-T Participants **eTable 4.** Feature Access Among QuitGuide Participants **eFigure.** Study Timeline

Supplement 3.
Data Sharing Statement
