# Supplementary material for: Just-in-Time Adaptive Intervention for Smoking Cessation in Low-Income Adults: A Randomized Clinical Trial
Source: JAMA Netw Open. 2025 Aug 14;8(8):e2526691. doi: 10.1001/jamanetworkopen.2025.26691 (PMC12355279; doi:10.1001/jamanetworkopen.2025.26691)
Supplement: Supplement 1. — Trial Protocol and Statistical Analysis Plan [file jamanetwopen-e2526691-s001.pdf]

**The University of Oklahoma Health Sciences Center**

**Study Protocol**
**Institutional Review Board**

**Title of Project:** Smartphone Based Smoking Cessation Intervention

**Principal Investigator:** Michael S. Businelle, Ph.D.; Department of Family and Preventive

**Co-Investigators:** Darla E. Kendzor, Ph.D.; Department of Family and Preventive Medicine
Damon J. Vidrine, Dr.PH; Department of Family and Preventive Medicine
Summer Frank, Ph.D.; TSET Health Promotion Research Center, OUHSC
Sara Vesely, Ph.D.; OUHSC College of Public Health
Emily Hebert, Dr.PH; TSET Health Promotion Research Center, OUHSC

**Abstract**
Smoking is becoming increasingly concentrated among individuals with the lowest levels of income, education, and occupational status. In fact, smoking rates in the United States among people living below the poverty line is nearly twice as high as those above the poverty threshold. Highly flexible and low burden technology-based treatment approaches may overcome many of the barriers that have limited the use and effectiveness of traditional smoking cessation treatments among low SES adults. Ecological momentary assessment (EMA), in which mobile devices are used to capture moment-to-moment experiences, allows for the measurement of phenomena in real-time within natural settings. Smartphone-based smoking cessation apps could offer easily accessible, highly tailored, and intensive interventions at a fraction of the cost of traditional smoking cessation counseling. The Smart-T app uses a lapse risk estimator to identify moments of heightened risk for lapse, and the algorithm tailors treatment messages in real-time based upon level of imminent smoking lapse risk and currently present lapse triggers.

**A. Specific Aims**

The primary long-term objective of this research is to reduce smoking rates among low SES adults. The next step towards this goal is to use a fully powered randomized clinical trial to compare the efficacy of the Smart-T app with the free and widely available National Cancer Institute (NCI) QuitGuide app for smoking cessation in the 26 weeks following a scheduled quit attempt. A total of 450 socioeconomically disadvantaged, treatment-seeking males and females will be randomly assigned to receive either the Smart-T intervention app or the QuitGuide intervention app. All participants will receive nicotine replacement therapy and complete EMAs, but only the Smart-T group will receive intervention messages that are tailored to the current level of lapse risk and current lapse triggers. The study aims are to:

1. **Determine the impact of treatment condition (i.e., Smart-T vs. QuitGuide app; n=225 per group) on biochemically verified smoking abstinence.**
   *Hypothesis 1:* Participants randomly assigned to Smart-T will have significantly higher rates of biochemically-verified abstinence than those assigned to QuitGuide at 26 weeks post-quit.
2. **Determine the effect of Smart-T treatment messages on key lapse risk factors.**
   *Hypothesis 2: S*moking urge, stress, and cigarette availability will decline more and cessation motivation will increase more following tailored messages that target these lapse



Human Research Participant Protection The University of Oklahoma IRB APPROVED AAHRPP
IRB NUMBER: 9860
IRB APPROVAL DATE: 06/23/2023

triggers in real-time (Smart-T condition) compared with similar situations wherein no messages are provided (QuitGuide condition).

## B. Background and Significance

Smoking is the leading cause of preventable death in the U.S.[1,2] and is implicated in an estimated 30% of all cancer deaths in this country.[3] Recent studies indicate that smokers have average life expectancies that are 11-12 years shorter than those who have never smoked.[4] Importantly, depending on the age that one quits, ex-smokers live up to a decade longer than those who do not quit.[4] Although smoking prevalence has declined to 13.9% among U.S. adults who are not living in poverty, 26.1% of those living in poverty are current smokers.[5] The prevalence of both poverty (16.6%)[6] and smoking (21.1%)[7] are greater in Oklahoma than in the US overall. More than a third of Oklahomans live in rural areas,[8] and rural residence is associated with elevated smoking prevalence.[9,10] Finally, Oklahoma is home to a large population of American Indians (12.9% of all residents),[11] who have higher smoking prevalence (29.2% smoke) than other racial/ethnic groups.[12]

Lower SES individuals are the fastest growing group of smartphone owners in the U.S. In fact, smartphone ownership has nearly tripled since 2011[13] to 67% in households earning less than $30,000 per year.[14,15] Furthermore, our recent work (August 2016; N=610) indicated that smartphone ownership is high even among homeless adults (56.1% have active smartphones).[16] Smartphone-based smoking cessation apps could play a significant role in improving cessation rates for current and future generations of lower SES smokers. Smartphone apps could offer secure (e.g., data can be encrypted), easily accessible (i.e., smartphone owners report that their phones are within arm's reach for 90% of waking hours[17]), highly tailored, and intensive interventions at a fraction of the cost of traditional smoking cessation counseling; thereby, overcoming many of the barriers that have hampered use of other empirically supported smoking cessation treatments among lower SES individuals.[18,19] Furthermore, after being downloaded, smartphone apps can deliver tailored content (e.g., video, text) even when cellular reception and/or minutes/data are not available, overcoming some of the limitations of quit lines and texting based smoking cessation interventions(see[20,21]).

Although there are hundreds of publicly available smoking cessation apps, few have been empirically evaluated using a randomized controlled trial design. In a content analysis of 252 available iPhone and Android apps for smoking cessation, Abroms and colleagues[22] found that very few apps adhered to proven strategies for smoking cessation (e.g., suggesting use of effective medications, connecting to quit lines or clinics).[22] QuitGuide is a free smartphone app developed by the Tobacco Control Research Branch at the National Cancer Institute (NCI) and based on content from Smokefree.gov.[23] Unlike most available apps, QuitGuide's content adheres to established clinical practice guidelines,[24] and includes features such as using motivational messages to encourage users to make a quit attempt, and detailed information about medications (e.g., nicotine replacement therapy; NRT). QuitGuide also enables and encourages users to track their mood, cravings, triggers, and lapses, and users can tag locations to receive support messages when they are in places they have deemed high risk for lapse. To date, only one study has examined the efficacy of the QuitGuide app. Bricker et al. compared QuitGuide to a smartphone delivered Acceptance and Commitment Therapy-based app (SmartQuit) in a randomized controlled trial.[25] The overall quit rate for participants who were randomized to QuitGuide was 8%, compared with 13% for SmartQuit. Thus, there appears to be much room for improvement in phone-based treatments.

Ecological momentary assessment (EMA), in which handheld devices (e.g., smartphones) are used to capture moment to moment experience, allows for the measurement of phenomena

Human Research Participant Protection The University of Oklahoma IRB APPROVED AAHRPP
IRB NUMBER: 9860
IRB APPROVAL DATE: 06/23/2023

in real-time within natural settings.[26,27] EMA data may facilitate a better understanding of the mechanisms involved in successful cessation attempts, those affecting smoking lapses, and those implicated in the transition from lapse to relapse. Our research team and others have identified momentary predictors of smoking cessation and smoking relapse (e.g., smoking urge,[28-31] stress,[29,31-34] easy cigarette availability,[31,32,35,36] interacting with people who are smoking,[30,31,36] alcohol use,[30-32,36] cessation motivation[29,31]). **These findings suggest that momentary changes in key variables may be tracked and potentially used to initiate interventions as they are needed.** Using smartphones to detect high relapse risk situations and automatically deliver tailored smoking cessation interventions may help socioeconomically disadvantaged smokers to avoid lapse and successfully quit smoking.

In summary, socioeconomically disadvantaged adults are less likely to quit smoking and are disproportionately affected by smoking-related morbidity and mortality.[37-40] EMA studies have identified social, psychological, and environmental triggers that contribute to smoking lapse.[31,32,36,41] To date, no interventions besides our recent pilot work (see **Preliminary Studies** section), have used EMAs to repeatedly assess current smoking lapse risk and automatically deliver tailored treatment content. Our preliminary work indicates that smartphones may be used to deliver automated, tailored, low burden, and easily accessible interventions to socioeconomically disadvantaged smokers, a population with substantial barriers that have hampered the use of traditional smoking cessation treatments. The proposed study would extend our previous work and determine if automated treatment content (e.g., messages focused on coping with lapse triggers, smoking cessation medication reminders) that is tailored to current situations can attenuate lapse risk.

## C. Preliminary Studies/Progress Report

Drs. Businelle and Kendzor have used smartphones in 9 studies to collect EMA data for up to 6 months and influence health behavior in high-risk samples, including homeless (e.g., 1R01MD010733-01A1; PI Businelle)[29,42] and other socioeconomically disadvantaged adults (e.g., R01CA197314-01; PI Kendzor).[31,34,43-45] In *Project Prevail*, EMA data were collected from smokers seeking treatment at a safety-net hospital tobacco cessation clinic (*N*=146).[34,44,45] Participants completed 83% of all EMAs. Using just 6 EMA variables (i.e., urge, stress, cigarette availability, alcohol use, motivation to quit, proximity to others smoking), we created a smoking lapse risk estimator that identified 80% of all smoking lapses within 4 hours of the lapse (false positive rate = 17%; see a detailed description of the development of the risk estimator here [31]). In another study, we developed the *Smart-T* app that includes on-demand features (e.g., tips on coping with stress) and incorporates the Prevail lapse risk estimator to deliver tailored messages based on a person's momentary risk for smoking lapse. We examined the feasibility of the Smart-T app among smokers recruited from an urban safety-net hospital tobacco cessation clinic (n=59). In that study, socioeconomically disadvantaged participants completed 87% of all EMAs and received an intensive level of tailored smoking-cessation treatment messages (102 messages on average) over a 3-week intervention period. We found that the on demand app functions were used regularly and the app was well-liked (e.g., 97% would like to use the app in the future if they were to relapse, and 85% would refer their friends who smoke to use the app).[31] Analyses of EMA data revealed that urges to smoke were significantly reduced when tailored urge messages were delivered by the app, as compared to instances where other types of messages were delivered ($p<.001$).[46] We also found reductions in stress ($p=.021$) and cigarette availability ($p<.001$) following tailored stress and cigarette availability messages, compared to other types of messages.[46] A total of 20% of participants were biochemically confirmed abstinent 12 weeks post-quit,[47] which is better than many interventions conducted with low SES smokers.[25,48-52] Finally, Dr. Businelle is completing a 3-armed pilot randomized


Human Research Participant Protection The University of Oklahoma IRB APPROVED AAHRPP
IRB NUMBER: 9860
IRB APPROVAL DATE: 06/23/2023

clinical trial (i.e., Project Smart-T2; OUHSC IRB #7195) that compares the Smart-T2 app to standard in-person smoking cessation clinic care and the free NCI QuitGuide app. Smokers who attend a clinic based tobacco cessation program are randomized to groups and followed for 13 weeks (1 week pre-quit through 12 weeks post-quit). All participants are asked to complete EMAs on study provided smartphones for 5 weeks. To date, 64 participants have reached the 12-week post-quit follow-up visit. A total of 26% of Smart-T, 14% of QuitGuide, and 21% of in-person participants have achieved biochemically confirmed 7-day point prevalence abstinence at the 12-week post-quit visit.

**D. Research Design and Methods**

**Eligibility Criteria.** Participants (N=450) will be recruited through the TSET Health Promotion Research Center (HPRC) Tobacco Treatment Research Program (TTRP) which is directed by Dr. Darla Kendzor (Co-I). Participants will also be recruited through advertisements (e.g. Facebook and Craigslist ads). Individuals seeking smoking cessation services at the TTRP will be given a verbal description of the study during their first visit, and those interested will be screened for study eligibility. Those who respond to study advertisements will complete a brief REDCap screener online (i.e., OKSmokerstudy.com), and those who qualify for the study will contacted by study staff and scheduled for a screening/baseline visit. Consistent with our previous research with low SES populations,[31,45,47,53-55] we have limited exclusion criteria so that the sample is as broad and representative of the population as possible. Interested individuals will be included in the study if they: 1) earn a score ≥ 4 on the Rapid Estimate of Adult Literacy in Medicine-Short Form (REALM-SF)[56] indicating > 6$^{th}$ grade English literacy level (required to complete EMAs; at least 88% of all individuals screened for our EMA studies have met this inclusion criterion), 2) are willing to quit smoking 7 days after the baseline assessment, 3) are ≥ 18 years of age, 4) have an expired CO level ≥ 7 ppm suggestive of current smoking for those who attend in person baseline visits or substantiate smoking status (e.g., produce a pack of cigarettes) during baseline assessments by sending picture of cigarettes to staff 5) are currently smoking ≥ 5 cigarettes per day, 6) have no contraindications to using NRT, 7) agree to complete EMAs and CO tests on a study provided or personal smartphone, 8) have household income ≤ 200% of the federal poverty guideline,[57] and 9) agree to complete the 26-week post-quit follow-up assessment over the phone and via EMA. We will ask participants to verify their mailing address by showing (e.g., texting) a member of our study team a picture of their photo ID or a photo ID with a bill that was mailed to participant to verify their address. Those who are unable to provide a valid photo ID or piece of mail with their name and address will not be eligible to participate in the study. During the screener, a trained research assistant will confirm the participant's residency status, date of birth, University of Oklahoma employment status, and social security number with the participant. To be in compliance with IRS regulations, the University of Oklahoma must collect residency status for all participants. This determines how the payment is handled. All payments required to be reported as outlined in this policy require collection of the participant's name, tax identification number (SSN or ITIN), address, amount paid and source of funding for the payment.

**Participant Flow.** Individuals who provide informed consent and meet the study inclusion criteria will complete the baseline assessment in TTRP clinic space (the TTRP is located in Research Parkway in a building that is adjacent to the PI's office) or via phone calls with study staff.

Following the baseline assessment, participants will be given instruction on how to complete phone-based EMAs and carbon monoxide assessments. All participants, regardless of


Human Research Participant Protection The University of Oklahoma IRB APPROVED AAHRPP
IRB NUMBER: 9860
IRB APPROVAL DATE: 06/23/2023

condition, will be compensated for completing the baseline assessments, phone based follow-up, and EMAs. In addition, participants will complete expired breath carbon monoxide tests using the portable iCO device 3 days per week (weeks 1-27).

**Study Overview.** Eligible and interested individuals will be randomized to 1 of 2 groups: 1) Smart-T smoking cessation intervention plus NRT, or 2) NCI QuitGuide smoking cessation intervention plus NRT. Stratified randomization will be used to assign participants to groups based upon race, sex, and cigarettes smoked per day. Participants will be followed for 27 weeks and will attend either 1 in-person visit or complete phone-based baseline visits. Participants will be prompted to complete daily EMAs and tests of smoking status using a portable, low-cost, carbon monoxide monitor (Bedfont iCO Smokerlyzer) on 3 days each week. This data will be used to address primary (i.e., biochemically verified 7-day point prevalence abstinence at 26 weeks post-quit) and secondary outcomes (e.g., 30-day point prevalence abstinence, number of days to first lapse, longest period of smoking abstinence). Geographic location data will be captured multiple times per day to link characteristics of the present environment (e.g., proximity to tobacco outlets) with cessation-related outcomes. We have already created software that integrates the iCO sensor into our Insight™ mobile application platform.

Participants who complete the in-person baseline visit will: 1) receive combination NRT (i.e., nicotine patch and gum/lozenge), and 2) complete EMAs daily on study provided smartphones. Participants who complete the remote baseline process will be mailed NRT, and a phone (if necessary) to complete EMAs. NRT has been shown to increase successful smoking cessation,[58] and is currently offered via Quitline in all but 5 states.[59] Combination NRT (patch + gum or lozenge) is considered a first line treatment,[60] is relatively low-cost, can be kept on-site for easy distribution to study participants and is more effective than the patch or gum/lozenge alone.[58] Consistent with over-the-counter package labeling, participants will be offered up to 10 weeks of nicotine patches (i.e., label dosage recommendations will be followed) and up to 12 weeks of nicotine gum or lozenges (i.e., participants will receive a 4 week supply of NRT at the completion of the baseline process and will be mailed NRT upon request for post-quit weeks 512).

Participants who own an Android smartphone (OS version 6.0 or higher) will be encouraged to download and use the study app on their own phone. Those who do not own this type of smartphone and those who do not have a data plan will be loaned a Samsung smartphone (or equivalent) for 27 weeks so that they may complete EMAs. Participants will navigate through the EMA program and enter data simply by touching the screen. The study app (i.e., Smart-T app or EMA only app) includes a “Call Staff” function/button that automatically calls study staff (e.g., if they have problems completing EMAs). The EMA app also includes a “Payment” button which, when pressed, indicates the number of EMAs that have been prompted and completed and the current level of compensation based upon the up-to-the-moment percentage of EMAs completed. Smartphones will automatically collect data when on-demand features are accessed for both the QuitGuide and Smart-T groups (e.g., number of times features are used). Use of personal vs. study provided phones will be examined as a covariate in all analyses.

Human Research Participant Protection The University of Oklahoma IRB APPROVED AAHRPP
IRB NUMBER: 9860
IRB APPROVAL DATE: 06/23/2023

Sub-Study: A sample (n=30) of American Indian/Alaska Native participants who have completed the 6 month follow-up assessment will be asked to participate in another qualitative interview designed to assess the perceived utility and cultural fit of the Smart-T App and NCI QuitGuide app. Information gathered from these qualitative interviews will be used to culturally modify and then experimentally test a new, culturally tailored, version of the apps for AI/AN smokers who want to quit smoking. In addition, this will serve as an incredibly valuable training opportunity for an emerging Native American scientist to learn tobacco cessation, mHealth, and qualitative methods research from an interdisciplinary team of leading experts in the field.

**Description of Treatment Groups.**

***NCI QuitGuide App + NRT Condition*** The National Cancer Institute's QuitGuide app is a free smartphone app that is available on the Apple and Android app stores and has been downloaded by thousands of users. QuitGuide is one of the few apps that includes many of the recommendations detailed in the Clinical Practice Guideline.[24] Individual level app feature use is accessible by request from the Smokefree.gov server (the PI has already obtained this type of QuitGuide data for a pilot study). The QuitGuide app aims to help smokers understand their smoking patterns and develop the skills needed to quit smoking. Participants who are randomly assigned to the QuitGuide condition will download the QuitGuide app and it will be set for a quit date 1 week following thebaseline visit. QuitGuide provides content during the pre-quit period to prepare participants for their quit attempt. Participants will receive information on how to use QuitGuide app features. During the baseline process and will be encouraged to use the app for the duration of the study. QuitGuide features are listed in Table 1. Participants will be instructed to click "I Was Smoke Free Today" and the "I Slipped" tab to update their status every day (see Figure 1). Participants can use the on-demand "Track My Craving" tab to record and get help with managing cravings. They can click the "Manage My Mood" tab to track their mood and get tips on managing bad moods.

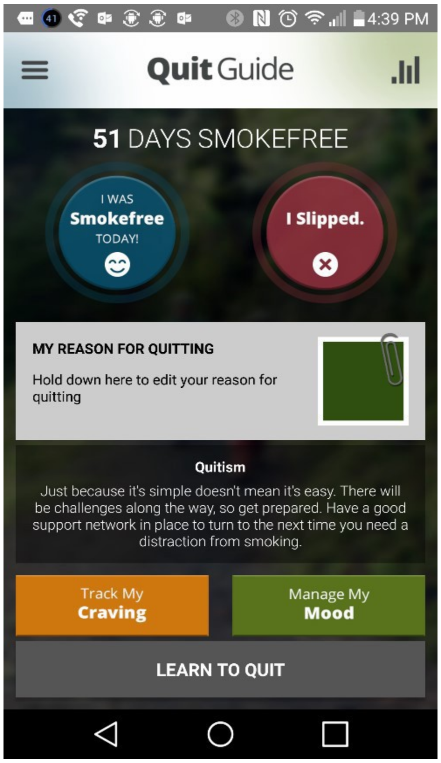


**Figure 1.** QuitGuide app home screen

Participants can also personalize the app home screen with their list of reasons for quitting. Clicking the icon on the top right of the home screen will open a summary screen that will help participants to monitor their smoking cessation milestones and money saved by not smoking. Clicking the icon on the top left of the home screen enables modification of the quit plan; setting places and times of day when/where participants would like to receive app messages, and create journal entries. Participants can also click "My History" to examine their mood, urge, and lapse history. Lastly, participants may click "How to Quit" to learn more about ways they can improve their chances of successfully quitting (Figure 1).

IRB NUMBER: 9860
IRB APPROVAL DATE: 06/23/2023

***Smart-T + NRT Condition.***

Participants who are randomly assigned to the Smart-T condition will download the Smart-T app and it will be set for a quit date 1 week following the baseline process. Smart-T provides content during the pre-quit period to prepare participants for their quit attempt. Participants will receive information on how to use Smart-T app features at the baseline visit and will be encouraged to use the app for the duration of the study. The Smart-T app contains multiple components including an EMA delivery and data transfer system, automated messages, and on demand content. Each feature is described below.

*Automated messages.* Over 400 unique messages have been created for the Smart-T app and message content has been evaluated by 5 tobacco treatment experts. Similar to our pilot study of the Smart-T app, 4 levels of automated messages will be used for this study (see Table 2). One message will be delivered at the end of each EMA (note: the message will include the participant's first name). Level 0 messages will be pushed during the one week pre-quit period. These messages are not tailored, but rather are delivered in a predetermined order. Message topics are primarily motivational and/or focused on planning and preparing for the quit attempt and benefits of quitting (e.g., "John, Now is the time to create a "smoke free" home environment! Studies show that people who ban smoking in their home increase their chances for quitting"; "John, Sometimes people save a pack of cigarettes (or even a few cigarettes) "just in case" or to prove they have the willpower to not smoke. DON'T! This just makes it easier to start smoking again!"; "John, it's OK to have mixed feelings about quitting. Don't let that stop you! There will be times that you don't feel like quitting! Stick with it anyway!"). Participants will receive level 4 messages during the post-quit period when they indicate that they smoked that day. Level 4 messages attempt to motivate the participant to stop smoking.

**Table 1.** Comparison of treatment conditions.

| Components | Smart-T | QuitGuide |
|---|---|---|
| Combination NRT | X | X |
| EMA | X | X (add on for this study) |
| Set a quit date | Quit date is set to 1 week after the baseline visit | |
| Smoking cessation psychoeducation | X | X |
| Treatment messages tailored to currently present lapse triggers | X | |
| On demand tips and exercises | | X |
| Coping with cravings | X | X |
| Coping with mood | X | |
| Coping with stress | X | |
| Coping with others smoking | X | X |
| Motivational messages | X | |
| Benefits of quitting | X | |
| Harms of smoking | X | |
| Coping with insomnia | X | |
| Coping with pain | X | |
| Scheduled cessation tips | | X |
| Individualized quit plan | | X |
| Record craving/withdrawal | X | X |
| Record slip | X | X |
| Record smoking triggers | | X |
| List reasons for quitting | | X |
| Summary of savings from smoking fewer cigarettes | | X |
| Share quit information on social media | | X |

**Table 2.** Smart-T message types.

| Type | Conditions for message delivery |
|---|---|
| Level 0 | Pre-quit |
| Level 1 | Post-quit when imminent lapse risk is low. |
| Level 2 | Post-quit when lapse risk is high & following lapse when participant is still interested in quitting. |
| Level 3 | Post-quit immediately following lapse. |
| Level 4 | Post-quit when already smoked that day. |

Human Research Participant Protection The University of Oklahoma IRB APPROVED AAHRPP
IRB NUMBER: 9860
IRB APPROVAL DATE: 06/23/2023

At the end of each post-quit EMA, participants will receive individually tailored (i.e., the message will include the participant's first name) automated messages based on their risk for imminent smoking lapse (see [31] for a complete description of our EMA based lapse risk estimator). Level 1 messages will be delivered when EMA responses indicate a low level of imminent smoking lapse risk, and message content will focus on maintaining abstinence motivation and general cessation advice (e.g., seeking social support for cessation, coping with and avoiding various lapse triggers, and benefits of quitting). Level 2 messages will be delivered when EMA responses indicate high imminent risk of smoking lapse OR the participant already smoked that day or the day before OR the participant indicates that they have a greater than 0% chance of smoking that day. Level 2 messages will also be delivered at the end of participant initiated About to Slip EMAs, Already Slipped EMAs, and when the iCO indicates recent smoking. Level 2 messages primarily focus on ways to cope with current lapse triggers (i.e., reported during the current EMA) and are tailored to the highest rated current lapse trigger (i.e., low motivation for cessation, high urge to smoke, high stress, easy access to cigarettes). In instances where multiple triggers are equally highly rated, one message will be delivered with preference given in the order listed above. The justification for this ordering system is grounded in the results from Projects Prevail and Smart-T. Specifically, preference is given to variables that were better at distinguishing moments of high risk for imminent lapse from moments of low risk for lapse. An innovative feature of this study is that when risk for lapse is determined to be high and a level 2 message is pushed, a second message will suggest that the participant use nicotine replacement (i.e., nicotine gum/lozenge) in that moment (see Figure 2). Level 3 messages will be delivered immediately after lapse occurs and these motivational messages will encourage a return to abstinence (e.g., "John, A slip is a sign that you need to improve your smoking cessation plan. Think about what went wrong and develop a stronger plan to stay quit. Keep trying and YOU WILL SUCCEED!"). *On-demand content.* Several Smart-T components are available through the smartphone app 24 hours a day, 7 days a week (see Smart-T pre-quit home screen in Figure 3). First, the "Call Counselor" function/button is programmed to automatically call the free Oklahoma Tobacco Help Line (1-800-Quit-Now) so that participants can reach a live counselor at any time (the Smart-T app automatically saves the duration of each Help Line call for future analysis).

**Figure 2.** Level 2 messages.

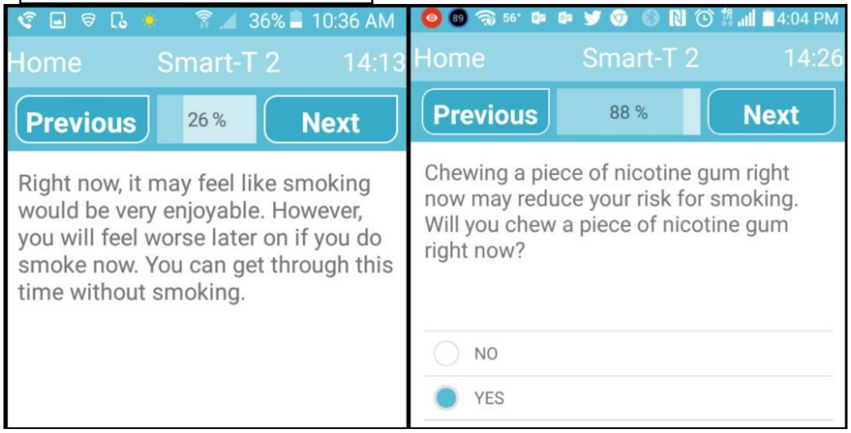


Second, the "Quit Tips" function/button links to a menu of treatment related messages (see Figure 4). Third, the "Medications" function/button offers information (e.g., common side effects, quit statistics, use instructions) about NRT medications that will be provided to participants (i.e., nicotine patch and gum/lozenge; see Figure 5).



IRB NUMBER: 9860
IRB APPROVAL DATE: 06/23/2023

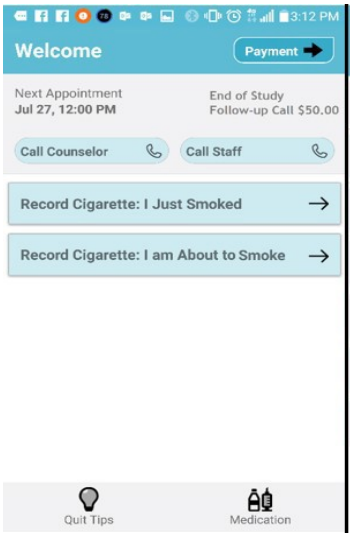


Figure 3. Smart-T pre-quit home screen.

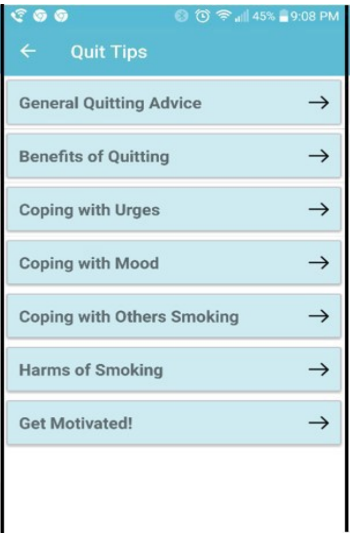


Figure 4. Quit Tips function.

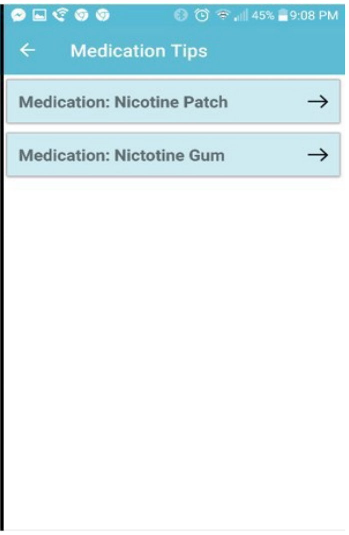


Figure 5. Medication function.

In the Smart-T feasibility study, most participants (83%) used the on-demand Quit Tips and Medication Tips features and participants viewed an average of 31 (median=18) Quit Tips and 15 (median=10) Medication tips per viewing session.[47] Participants may have viewed such a high number of tips because they found the information useful and engaging. High participant ratings for helpfulness/ usefulness of the on-demand Tip features adds weight to this explanation.[28]

*Other Smart-T app features.* During the pre-quit period, Smart-T participants will receive a daily message that reminds them of the number of days until their scheduled quit date (e.g., "John, You are scheduled to quit smoking in 5 DAYS at 10:00 pm next Sunday night. Developing a plan to quit and taking your medications will GREATLY increase your chances for staying quit."). Periodically, participants also receive messages to remind them that the on demand features are available when/if needed.

**Description of Study Measures.**

***Baseline and Follow-Up Assessments.*** Measures will assess demographic and smoking characteristics, multiple constructs that are known to be related to lapse (e.g., depression, stress, affect, social support), and document intervention effects (See Table 3 and Appendix). We will also access participant perceptions of each app feature. The Baseline process will take approximately 60-90 minutes to complete and data will be collected either on laptop/tablet computers using Questionnaire Development System (QDS) software or remotely via REDCap surveys. QDS uses a computer-administered self-interview format (i.e., ACASI), which reduces data entry errors and the need to retain paper copies of raw data. Each item appears on the computer screen while the program simultaneously reads the item (participants touch the screen to select answers only after QDS reads each item). Participants wear headphones so that others do not hear the survey items. Participants from our previous studies in similar populations, including those with no computer experience, have reported few problems using the QDS program. Staff will be available to help participants who may have difficulty. The REDCap surveys have been designed to replicate the QDS version as closely as possible. Up to 30 participants will complete a 16-week qualitative interview with a staff member over the phone regarding their experiences in the study so far. The 26 week post-quit follow-up assessment, which includes a longer EMA (i.e., 20-30 minutes) will be completed by participants in the smartphone application or through REDCap survey. Additionally, up to 100 intervention group participants and up to 100 control group participants will complete a 15-30 minute



Human Research Participant Protection The University of Oklahoma IRB APPROVED AAHRPP
IRB NUMBER: 9860
IRB APPROVAL DATE: 06/23/2023

qualitative interview with a staff member over the phone (or via REDCap if a medical condition

prevents them from being able to speak for long periods of time) as part of their 26 week post-quit follow-up assessment. Recordings will be transcribed by internal staff, Microsoft Transcribe, or by approved contractors with whom a Business Associates Agreement has been made. When using Microsoft Transcribe, all transcriptions will be reviewed by study staff. Participants will be notified that they will earn a $5 bonus if they complete the final EMA within the first 24 hours that it is available.



Human Research Participant Protection The University of Oklahoma IRB APPROVED AAHRPP
IRB NUMBER: 9860
IRB APPROVAL DATE: 06/23/2023

***Ecological Momentary Assessment (EMA)*** enables measurement of phenomena in real-time, natural settings.[26,27] EMA items will identify fluctuations in key variables that predict study outcomes with less bias than traditional in-person assessments. <u>EMA data will be used to identify moments of high smoking lapse risk, tailor Smart-T messages, and examine treatment mechanisms.</u>

**<u>EMA types.</u>** The EMA methodology used in this study will be similar to what has been used in our previous studies, and by other researchers.[29,31,36,43,47,63,76-80] EMA items will assess multiple

**Table 3.** Measures.

| **MEASURE** | **ITEMS** | **FULL BATTERY ASSESSMENTS** | | | **EMA**[31,36,47,61-63] |
|---|---|---|---|---|---|
| | | **Screen** | **Base** | **26-W** | **Morning Daily Diary‡** |
| Screening Questionnaire | 22 | X | | | Cigarettes Smoked |
| Rapid Estimate of Adult Literacy in Medicine-Short Form[64] | 9 | X | | | Social Interactions and support |
| Weight/Height/CO | 5 | | X | | NRT Use |
| Smoking Status Questionnaire[65] | 2-4 | | 2 | 4 | Side Effect Items |
| Tobacco History Questionnaire | 40 | | X | 20 | Alcohol Consumption |
| Heaviness of Smoking Index[66] | 2 | | X | X | Sleep Items |
| Self-Efficacy Scale[67] | 10 | | X | X | |
| Demographic/Background Questionnaire | 23 | | X | | **End of Day Daily Diary‡** |
| Scale of Subjective Social Status | 2 | | X | X | Cigarettes Smoked |
| Urban Life Stressors Scale[68] | 21 | | X | | Alcohol + Marijuana Use |
| Center for Epidemiological Studies Depression[69] | 10 | | X | X | iCO Breath Test (3 times per week) |
| Perceived Stress Scale[70] | 4 | | X | X | Weekly App Questions |
| Pandemic Stress Index (PSI) [95] | 3 | | X | X | **Random/Event EMAs** |
| Interpersonal Support Eval. List[71] | 12 | | X | | |
| Self-Rated Health[72] | 31 | | X | 10 | Recency of Smoking |
| BRFSS Inadequate Sleep | 5 | | X | | Urge to Smoke |
| BRFSS Adverse Childhood Experience | 11 | | X | | Stress |
| Health Related Quality of Life | 3 | | X | X | Availability of Cigarettes |
| Patient Health Questionnaire + GAD7 | 15 | | X | X | Recent Alcohol Use |



Human Research Participant Protection
The University of Oklahoma
IRB APPROVED
AAHRPP
IRB NUMBER: 9860
IRB APPROVAL DATE: 06/23/2023

constructs that are hypothesized to be related to smoking lapse (see Table 3). Three types of EMAs will be used: daily diary, random sampling, and event sampling. Random and daily diary EMAs will be prompted and initiated by the phone. The phone will ring and vibrate to cue these EMAs for 30-60 seconds. If the participant has not responded after 5 prompts, the EMA will be recorded as missed. Event sampling is initiated by participants. Participants are instructed to self-initiate "Record Cigarette" EMAs during the pre-quit period, and "Lapse" EMAs during the post-quit period. We expect random and event sampling EMAs to take approximately 30 seconds to complete and daily diary EMAs to take approximately 1-2 minutes to complete. All EMAs will be date and time stamped for future analyses.

*Daily diary.* Daily diary EMAs will be completed twice each day for the first 14 weeks of the study and once per day for the last 13 weeks of the study. The phone will initiate the morning daily diary 30 minutes after the participant's pre-set wake time (weeks 1-14) and questions ask about the previous day (e.g., "How many cigarettes did you smoke yesterday?" "Did you use marijuana yesterday?," and "Did you drink any alcohol yesterday?" If the participant answers "yes," he/she will be prompted to indicate the type and quantity of the alcohol/cigarettes that were consumed). In addition, participants will be asked each day if they experienced severe NRT side effects (the app will instruct participants to call 911 if they experience severe NRT side effects, and it will automatically send research staff an encrypted email to contact participants). For the entire 27 weeks of the study, the end of day daily diary will be completed 60 minutes before the

| | | | | | |
|---|---|---|---|---|---|
| Alcohol Quantity and Frequency Questionnaire[73] | 9 | | X | X | Cessation Motivation |
| Alcohol Quantity and Frequency Questionnaire[73] | 9 | | X | X | Interaction with Smokers |
| Treatment Quality and Satisfaction Survey | 19 | | | X | Social setting/Location |
| Treatment Quality and Satisfaction Survey | 19 | | | X | Reasons for Lapse* |
| Treatment Improvement Interview[74] | 18 | | | X | Cigarette Reward Value* |
| Locator Form[75] | 8 | | X | | Lapse Warning Signs* |
| Financial Strain Questionnaire | 9 | | X | | **Weekly EMA** |
| MacArthur Major Discrimination | 4 | | X | | Pain |
| Modified Cigarette Evaluation Measure | 12 | | X | | Treatment Quality |
| Short Scale Anxiety Index | 5 | | X | X | HRQoL |
| Religious Questionnaire | 2 | | X | | Readiness to Change |
| Perceived Neighborhood Disorder and decay | 15 | | X | | Sleep |
| Collective Efficacy | 10 | | X | | Smoking Items |
| Informal Integration with Neighbors | 3 | | X | | Pandemic Stress Index |
| Fear & Mistrust | 7 | | X | | Covid Vaccine |
| Personal Victimization | 3 | | X | | |
| Perceptions of Mobile Phone Intervention Questionnaire | 23 | | X | | |
| Covid Vaccine | 5 | | X | X | |
| Delay Discounting Task (TOAD) | 10 | | X | | |
| Time needed to complete assessment (minutes) | -- | 8 | 60 | 40 | ‡Note: Daily Diary EMAs also contain all Random EMA items |
| Note. *Event EMA's only. Screen = Screening; Base = Baseline; 26-W = 26Week Post-Quit Follow-Up. | | | | | |



Human Research Participant Protection The University of Oklahoma IRB APPROVED AAHRPP
IRB NUMBER: 9860
IRB APPROVAL DATE: 06/23/2023

participant's pre-set bedtime. If the participant does not respond to the daily diary assessments, they will be rescheduled (up to 2 times) 15 minutes later. A carbon monoxide test will be included as one component of this EMA 3 days each week. Participants will not receive credit for completing the end of day EMA if they do not complete the prompted iCO breath tests. Both the morning and end of day daily diary EMAs will include all random sampling items. Constructs that will be assessed are listed in Table 3 and actual EMA items are presented in the Appendix. Note: During one EMA each week, participants will be asked multiple questions about the usefulness of their app (see Appendix).

*Random sampling.* Participants will be prompted to complete random EMAs, scheduled to occur during each participant's normal waking hours, 3 times each day for 5 weeks (1 week pre-cessation and 4 weeks post-cessation) and 1 time per day for the next 9 weeks (week 6 through 14). Random sampling will not be conducted during weeks 15-27. EMA items will assess the 6 items used for the lapse risk estimator (i.e., urge to smoke, stress, recent alcohol use, interaction with other smokers, cessation motivation, and cigarette availability). Other relevant constructs will also be assessed during random sampling (e.g., recency of smoking, odds of smoking today; See Table 3 and Appendix).

*Event sampling.* All participants will be asked to self-initiate Smoking EMAs (pre-quit week), and Lapse EMAs (post-quit for 26 weeks), see Smart-T pre-quit home screen in Figure 3). Smoking EMAs. During the first week of assessment (pre-quit week), participants will be instructed to indicate when they smoke (by clicking the "Record Cigarette" button). Because the assessment burden would be excessive for heavy smokers if each smoking occasion were assessed, the smartphone will randomly sample up to two smoking occasions from each participant per day. Lapse EMAs. After the quit date, participants will be instructed to complete lapse EMAs (by clicking the "About to Slip" or "Already Slipped" buttons) when relevant. Lapse EMA items are nearly identical to those presented in the random EMAs. However, questions are worded to separately assess the participant's responses immediately prior to AND following the lapse. "About to Slip" EMAs are followed by a second EMA 15 minutes later to assess whether participants actually lapsed. Participants are instructed to complete an "Already Slipped" EMA when they lapsed, but did not complete the "About to Slip" EMA prior to the lapse. Post-lapse EMAs query about the reinforcing value of the lapse cigarette(s), and causes of the lapse. Constructs that will be assessed are listed in Table 3 and actual EMA items are presented in the Appendix.

The mHealth Shared Resource at the OUHSC and Stephenson Cancer Center (SCC) has developed the Insight™ mHealth Platform which empowers researchers to build, test, and launch technology-based assessment and intervention tools. The mHealth resource employs a program manager and 4 senior programmers who develop and maintain web and mobile applications and relational databases. Applications are developed using state-of-the-art cross platform design tools. The Resource is located within the TSET Health Promotion Research Center (HPRC), where Dr. Businelle's office is located. Dr. Businelle is the Scientific Director of the mHealth Shared Resource, which will provide the programming services for the proposed project.

*Smartphone training.* Participants will watch a brief step-by-step video tutorial (created by the researchers) at the baseline visit that demonstrates use of the app features (i.e., QuitGuide or Smart-T). Additional instructions on how to use the study apps will be loaded into the app home screen so that participants may view them at any time. We have achieved high EMA compliance rates (i.e., 82%-87% of all EMA's completed) using similar protocols in 5 samples of socioeconomically disadvantaged smokers (e.g., homeless smokers, safety-net hospital patients).[29,31,47,54]


Human Research Participant Protection The University of Oklahoma IRB APPROVED AAHRPP
IRB NUMBER: 9860
IRB APPROVAL DATE: 06/23/2023

*Data loss prevention.* In order to overcome potential loss of data if participants lose their phone (less than 1% of phones have been lost in most studies), phones will be programmed to connect to our secure server each day to upload encrypted data. This will ensure that no collected EMA data are lost. This tactic will also allow the researchers to remotely monitor each participant's EMA completion rate and intervene (e.g., call the participant) when this rate is low (calls will be noted in the dataset and this variable may be used as a covariate in study analyses). Importantly, EMA data are password protected and encrypted on the phone and only encrypted data are transmitted to our secure server. Thus, study data are ONLY accessible by the research team. If a phone is lost, the app and all study data will be remotely wiped and only one replacement phone will be provided to each participant.

*EMA alert settings.* At the baseline visit, a phone set-up wizard is used to set participant sleep and wake times for each day of the week (Note: sleep/wake times can be changed for those with variable schedules). This practice reduces the likelihood that the phone will ring when participants are sleeping. In addition, participants may delay random EMAs by up to 20 minutes (i.e., by clicking the snooze assessment option when an EMA is prompted). Daily diaries will be automatically rescheduled 15 minutes later (two times) if a participant does not respond to the prompt.

*Compensation.* All participants will be compensated $30 for completing the Baseline assessment and $50 for completing the Week 26 post-quit follow-up assessment (note: participants who complete the 26 post-quit follow-up EMA within 24 hours will receive a $5 bonus) and qualitative interview at either the 16-week mark or the 26-week mark. Participants will only be asked to complete 1 qualitative interview and will not be asked to complete both interviews. We will ask all participants to complete interviews until we have completed 30 16-week interviews and a combined 200 26-week interviews (100 per group). Participants will also be compensated monthly via GreenPhire gift cards based upon the percentage of Daily and Random EMAs that were completed. During the first 37 days (~5 weeks) of the study (participants will be prompted to complete 5 EMAs per day). In the first 7 days those who complete 50%-69% of all assessments will receive $12 in gift cards, those who complete 70%-79% of assessments will receive $18 in gift cards, and those who complete 90% or more of their EMAs will receive a total of $25 in gift cards. During the next 30 days, participants will earn up to $50 per 15-day period (i.e., ≥90% of EMAs completed = $50 per 15- day period, 80%-89% = $36 per 15-day period, 50%-79% = $24 per 15-day period). During the next 60 days (~9 weeks) of the study (weeks 6-14), participants will be prompted to complete 3 EMAs per day, and will earn up to $36 per 15-day period (i.e., ≥90% of EMAs completed = $36 per 15-day period, 80%-89% = $28 per 15-day period, 50%-79% = $18 per 15-day period). During the final 13 weeks of the study (weeks 15-27; participants will be prompted to complete 1 EMA per day), participants will earn up to $30 per 15-day period depending on the percentage of EMAs completed (i.e., ≥90% of EMAs completed = $30 per 15-day period, 75%-89% = $22 per 15 day period, 50%-74% = $14 per 15-day period). PARTICIPANTS WILL NOT BE COMPENSATED FOR ACCESSING ON-DEMAND APP FEATURES OR COMPLETING TREATMENT COMPONENTS. Participants will not receive payment for phone surveys until they confirm or update their contact information (i.e., participants can click a button in the app to confirm/update their contact information, or study staff will attempt to contact them every 15 days to update/confirm contact information). If an interview eligible participant does not complete the 26-week post-quit follow-up assessment (either the phone survey component or the phone call interview component), he/she will be contacted to reschedule and request that loaned phones be returned in person or by mail.

**Supplemental Qualitative Interview: Those who participate in the supplemental qualitative**

Human Research Participant Protection
The University of Oklahoma
IRB APPROVED
AAHRPP
IRB NUMBER: 9860
IRB APPROVAL DATE: 06/23/2023

**interview will receive additional $30 compensation. The supplemental qualitative interview will take approximately 20-30 minutes to complete.**



Human Research Participant Protection
The University of Oklahoma
IRB APPROVED
AAHRPP
IRB NUMBER: 9860
IRB APPROVAL DATE: 06/23/2023

**Biochemical Assessment of Smoking Status.** Consistent with most published smoking cessation RCTs (see Clinical Practice Guidelines[24]), our primary study outcome will be biochemically confirmed 7-day point prevalence abstinence 26 weeks following the scheduled quit date. Secondary outcomes (e.g., 30-day point prevalence, time to first lapse, longest time of quit) will also be examined. Smoking abstinence will be measured daily via EMA self-reports and the Bedfont iCO smokerlyzer will be used to verify smoking status on 3 days per week (during the end of day EMA). Participants will be prompted to connect the iCO device to the study smartphone and follow step-by-step directions to complete the iCO test (e.g., hold your breath countdown, exhale countdown; See Figure 8). Results of these tests will be date and time stamped and saved. The Smart-T algorithm will utilize the results of the iCO test to inform the feedback that is delivered at the completion of EMAs. Please note that we have integrated automated and secure (i.e., encrypted) facial recognition software to ensure that only the participant completes prompted iCO tests. Our CO criteria for abstinence is consistent with numerous studies using cutoffs of ≤ 6 ppm.[81-86] The half-life of CO is up to 8 hours depending on a variety of factors (e.g., time of day, daily smoking rate, recency of smoking, physical activity).[87] Studies have shown that expired CO is a valid indicator of smoking status and cessation outcomes and compares favorably with cotinine and other biochemical measures that have longer detection windows.[88-91] We believe that self-reports of abstinence combined with CO levels suggestive of recent abstinence provide an accurate, immediate, and practical measure of abstinence. Finally, the manufacturer indicates that the iCO is valid for approximately 200 CO tests and has a sensor sensitivity of 1 ppm.[92] Our protocol will require 81 CO testing sessions (3 per week for 27 weeks), allowing for repeated tests when/if needed.

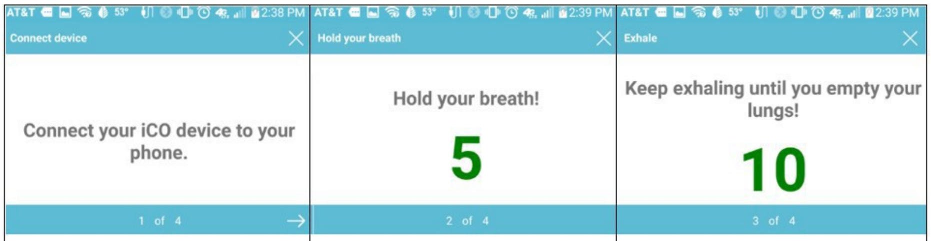


Figure 8. iCO collection steps.

**Description of Study Visits**

The Baseline visit will take approximately 60-90 minutes to complete and data will be collected on laptop/tablet computers using Questionnaire Development System (QDS) software. Staff will be available to help participants who may have difficulty. The Remote Baseline process will occur via phone calls with research staff, and data will be collected via REDCap surveys. Up to 30 participants will complete a 16-week qualitative interview with a staff member over the phone regarding their experiences in the study so far.

The 26 week post-quit follow-up assessment is a longer than usual phone based EMA that will take 20-30 minutes to complete. Up to 100 intervention group participants and up to 100 control group participants will complete a 15-30 minute qualitative interview with a staff member over the phone (or via REDCap if a medical condition prevents them from being able to speak for long periods of time) as part of their 26 week post-quit follow-up assessment. Constructs that will be assessed are listed in Table 3 and actual assessment items are presented in Appendix A.

## E. <u>Statistical Methods</u>

**Aim 1.** We will compare the proportion of participants that are biochemically verified as abstinent (i.e., iCO and self-reports indicate no smoking in the past 7 days) between the treatment conditions (i.e., Smart-T vs. QuitGuide), at 26 weeks post-quit using a chi-square or

Human Research Participant Protection
The University of Oklahoma
IRB APPROVED
AAHRPP
IRB NUMBER: 9860
IRB APPROVAL DATE: 06/23/2023

Fisher's exact test. We will perform the analysis under the intention to treat (ITT) assumption wherein participants who miss iCO EMAs will be considered not abstinent. We will examine the missing rate and missing patterns before analysis. If the combined missing rate is very small, say less than 5%, then we may safely perform the data analysis on the available data or the data assuming ITT. If the missing rate is high, then we will explore sequential multiple imputation (SMI) as described by Raghunathan et al.[93] The analysis results from SMI data will be compared with those from the available data and ITT analyses. Based upon previous studies of mobile phone based interventions for smoking cessation (see Cochrane Review[94]), we expect that 9% of those receiving the QuitGuide + NRT treatment will be abstinent at the 26 week post- quit follow- up visit, and based upon our pilot work, we expect that 18% of those receiving the Smart-T + NRT treatment will be abstinent at the 26 week post-quit follow-up visit (see[47]). With an alpha of 0.05 and 225 participants in each group, we have 80% power to detect a 9% difference in biochemically confirmed 7-day point prevalence abstinence at 6 months (i.e., 18% abstinent in the Smart-T group vs. 9% abstinent in the QuitGuide group). We expect that groups will be similar in baseline characteristics (e.g., sex, nicotine dependence) due to randomization; however, in addition to the chi-square analysis, we will perform logistic regression to determine if the odds of abstinence vary by other baseline characteristics (e.g., sex, race/ethnicity). Finally, we will examine the interaction between treatment condition and sex (and race) to identify potential differential effects on abstinence. The enrollment/baseline type (e.g., in person vs. phone call) will be included as a potential covariate in analyses.

We will examine agreement across the iCO and self-reported abstinence measures. Also, we will use the self-report and iCO data to construct secondary outcome measures, including 30day point prevalence abstinence, time to first lapse, and longest period of abstinence, which will be compared between treatment conditions. Assuming an 80% iCO compliance rate for each participant, we anticipate 62 iCO measures per participant over the study. Lastly, we will examine changes in abstinence rates over time using generalized linear mixed model (GLMM) regression analysis employing a random intercept or slope parameter, as appropriate, and modeling the covariance structure for the repeated outcome measures, while accounting for participant characteristics, such as sex.

**Aim 2.** To determine the impact of treatment condition (i.e., Smart-T vs. QuitGuide) on key lapse risk factors (i.e., smoking urge, stress, cigarette availability, and cessation motivation), we will examine the effect of tailored messages on lapse risk factors using consecutive pairs of EMAs in which the first EMA in the pair results in a tailored message. These paired EMA scores will be used to identify whether the change in the lapse risk factor rating from the first to the second EMA in the pair differs depending on whether the participant receives a tailored message or not (i.e., Smart-T vs. QuitGuide). Assuming that each participant will receive a message targeting a particular risk factor at least once, then we have greater than 80% power to observe an effect size of 0.27 or larger in the mean of the change in the score for that risk factor in the Smart-T group compared with the QuitGuide group. In the Smart-T feasibility study,[47] mean changes in raw scores for key lapse factors ranged from 0.19 to 0.56; all effect sizes were 0.27 or larger, therefore we have ample power to detect potentially clinically relevant changes. There will be up to four pairs of EMAs per day across weeks 2-14 of the study. Assuming an 80% EMA completion rate and 25% of EMA pairs being considered high risk (thus resulting in a message tailored to a specific high risk trigger), we estimate 73 high risk EMA pairs to examine per participant. Roughly 25% of EMA pairs should target each key lapse risk factor, resulting in 18 EMA pairs for each message type per participant. With 225 participants per group, that provides 4,050 EMA pairs per message type per group to include in the longitudinal analyses, resulting in ample power to identify the minimal effect size assumed to be clinically relevant. We will also examine the changes in key lapse risk factor scores

Human Research Participant Protection The University of Oklahoma IRB APPROVED AAHRPP
IRB NUMBER: 9860
IRB APPROVAL DATE: 06/23/2023

using linear mixed model (LMM) regression analyses employing a random intercept or slope parameter, as appropriate, and modeling the covariance structure for the repeated outcome measures, while accounting for baseline participant characteristics. Another analysis will look across all paired EMA scores within an individual and assign each individual a success percentage score based on the number of times their paired EMA scores decreased by at least one point on the Likert scale. For example in an individual that has 50 paired EMA scores for smoking urge, if 40 resulted in a decrease in urge, 5 resulted in no change, and 5 resulted in an increase in urge, the success percentage score would be 80% (40/50). Then we can compare the mean success percentage scores between treatment conditions using independent t-tests followed by regression analysis to account for baseline participant characteristics. Additional analyses will compare success percentage scores early versus late in the study to examine if the effectiveness of tailored messages change through time. Finally, we will examine the interaction between Smart-T message type and sex (and race) to identify potential differential effects on lapse risk factors.

**F. <u>Gender/Minority/Pediatric Inclusion for Research</u>**

<u>Inclusion of Women and Minorities</u>. The study has no inclusion/exclusion criteria based on gender or race/ethnicity. However, QDS and EMA data collection software is currently offered in English only; therefore, all participants must be able to read English at > $6^{th}$ grade level. Based on historical data from TTRP patrons, study participants are expected to be 59% White, 25% Black, 10% American Indian, 6% other race, 3% Hispanic, 61% female, and 45% will report < $20,000 in yearly household income. No eligible participants will be excluded from this study due to gender, race, or ethnicity. We will continuously monitor enrollment in order to ensure that we are meeting recruitment goals to avoid under-recruiting minorities. If the enrollment for minorities is lower than expected, we will make special efforts to increase participation by advertising in community newspapers, local church organizations, and community centers. We expect that intervention groups (i.e., Smart-T and QuitGuide) will be similar in baseline characteristics (e.g., sex, race/ethnicity) due to randomization; however, in addition to the chi-square analysis, we will perform logistic regression to determine if the odds of abstinence vary by baseline characteristics (e.g., sex, race/ethnicity). We will examine the interaction between treatment condition and sex (and race) to identify potential differential effects on abstinence. Finally, we will examine the interaction between Smart-T message type and sex (and race) to identify potential differential effects on lapse risk factors.

<u>Inclusion of Children</u>. Services provided by the TTRP (the proposed data collection site) are available to any person ≥ 18 years of age. Children < 18 years of age do not receive services at the TTRP (they are referred to the Oklahoma Tobacco Helpline) and will not be included in this study for several reasons. First, in order to include these children, the assessment protocols being tested would require significant modification to take into account their differing cognitive and psychosocial development. Making these modifications would result in a fundamentally different set of tasks and would essentially necessitate the conduct of two different studies. We do not consider this feasible, short of submitting two different grant applications. Second, the FDA has not approved the use of over the counter nicotine replacement therapy in children under the age of 18, so we would not be able to provide the full treatment to those individuals, making the use of the protocol less than ideal and no longer state-of-the-art. Third, modal quit behavior for smoking occurs in adulthood, making this developmental time period particularly well suited to a test of mediating processes during a smoking cessation intervention.

Human Research Participant Protection The University of Oklahoma IRB APPROVED AAHRPP
IRB NUMBER: 9860
IRB APPROVAL DATE: 06/23/2023

**G. Human Participants**

1. **Subject Population.** Participants (N=450) will be recruited through the TSET Health Promotion Research Center (HPRC) Tobacco Treatment Research Program (TTRP). Participants will also be recruited through advertisements (e.g. Facebook and Craigslist ads). Those who respond to study advertisements will complete a brief REDCap screener online (i.e., OKSmokerstudy.com), and those who qualify for the study will contacted by study staff and scheduled for a screening/baseline process. Interested individuals may be included in the study if they: 1) demonstrate > 6th grade English literacy level (i.e., phone based EMAs require >6th grade literacy; at least 88% of all individuals screened for our previous EMA studies have met this inclusion criterion), 2) are willing to quit smoking 7 days from the Baseline process, 3) are ≥ 18 years of age, 4) have an expired carbon monoxide (CO) level ≥ 7 ppm suggestive of current smoking, or substantiate smoking status (e.g., send picture of their pack of cigarettes) during the remote baseline process conducted via phone, 5) are currently smoking ≥ 5 cigarettes per day, 6) are willing and able to complete the 27 week follow-up session over the phone and via EMA, 7) are willing and able to complete EMAs and CO tests on their personal smartphone (those who use their personal smartphone will receive an additional $15 per month to offset data plan costs if at least 50% of assessments are completed and contact details are confirmed) or a study provided smartphone, 8) have household income ≤ 200% of the federal poverty guideline, 9) have no contraindications for over the counter nicotine replacement therapy (i.e., individuals with uncontrolled blood pressure, history of myocardial infarction within the past two weeks, or current pregnancy or plans to become pregnant during the study period will be excluded).

2. **Sources of Research Material.** All data collection will follow HIPAA guidelines. There will be 3 sources of data for the current study: 1) in-person or call based data collected during the baseline process. The Remote Baseline process will occur via phone calls with research staff, and Baseline data will be primarily collected via REDCap surveys, 2) EMA data collected on study-provided or personal smartphones via the encrypted Insight™ smartphone application, and 3) expired carbon monoxide data assessed via Vitalograph ecolyzer (Baseline visit only) and Bedfont iCO smokerlyzer (Baseline and EMA). Measurements of height and weight will be collected at the baseline visit.

3. **Recruitment and Informed Consent.** Individuals seeking smoking cessation treatment during their first TTRP visit will be approached by study research staff to discuss this study. Participants will also be recruited through advertisements (e.g. Facebook and Craigslist ads). All study staff have completed training in the ethical conduct of research (including CITI training). Prior to screening, potential participants will receive information about this voluntary study and will be informed that TTRP services are not contingent upon study enrollment. Those who remain interested in participating will be provided detailed information about the study by research staff, given the opportunity to have their questions answered in a private room or in phone calls, and will complete written informed consent via paper or REDCap. Remote participants will review and sign consent forms electronically via a REDCap link, and will be provided a copy for their records.

4. **Risks**. Participation in this study poses minimal risk to participants. Potential risks include loss of confidentiality, coercion related to compensation for completion of study assessments, nicotine withdrawal, and side effects from nicotine replacement therapy. Each of these risks are discussed below. The consent form will clearly describe the potential risks of this study. Thus, participants who feel uncomfortable with such assessments or intervention programs are not likely to agree to participate.

Human Research Participant Protection The University of Oklahoma IRB APPROVED AAHRPP
IRB NUMBER: 9860
IRB APPROVAL DATE: 06/23/2023

*Loss of confidentiality*. One potential risk is loss of confidentiality. The severity of harm in the case of loss of confidentially may range from mild to severe depending upon the individual and the specific circumstances. However, the risks of participation in the study are similar to that of participation in usual in person or phone based smoking cessation treatments, as loss of confidentially may be experienced in each case. Other possible adverse events might include compromised data security and discomfort related to being asked study questions. The research team will monitor risk and report adverse events immediately to the IRB. Because sensitive data will be collected, a Certificate of Confidentiality will be obtained prior to the onset of data collection to ensure that data cannot be subpoenaed and used against participants in court, and this information will be explained to all participants during the informed consent process (Certificates of Confidentiality are now automatically granted for all NIH grants).

*Compensation and Potential for Coercion*. Considering the very low-income levels of the anticipated study sample, we are aware of the potential for coercion in this study due to the provision of subject payments. We have paid particular attention the size of payments, considering the effort required to complete study assessments. Participants will receive a $30 gift-card for completing the baseline assessment and $50 gift-card for completing the 26-week post-quit assessment (those who complete the final longer EMA within 24 hours will receive a $5 bonus payment) and qualitative interview at either the 16-week mark or the 26-week mark. Participants will only be asked to complete 1 qualitative interview and will not be asked to complete both interviews. We will ask all participants to complete interviews until we have completed 30 16-week interviews and a combined 200 26-week interviews (100 per group). In addition, participants will receive compensation based upon the percentage of EMAs that they complete each month. Participants will be able to earn a maximum total of $624 in gift cards over 27 weeks (average of < $20/week).

*Nicotine Withdrawal*. There is a strong likelihood that most study participants who quit smoking will experience some nicotine withdrawal symptoms, including anxiety, restlessness, anger, irritability, sadness, problems concentrating, appetite change and weight gain, insomnia, and decreased heart rate. Withdrawal symptoms are usually short-lived, with most symptoms abating within 1-2 weeks. Importantly, Drs. Businelle and Kendzor are trained and experienced in addressing these issues as they emerge and the research team will be trained and supervised in the monitoring and addressing of psychological problems.

*Nicotine Replacement Therapy (NRT; Patch and Gum/Lozenge)*. Nicotine patch and gum/lozenge are smoking cessation medications that are available over the counter and side effects (e.g., nausea, skin irritation) are generally mild. All participants will receive NRT. NRT has been shown to increase successful smoking cessation, and is currently offered via Quitline via mail in all but 5 states. Consistent with over-the-counter package labeling, participants will be offered up to 10 weeks of nicotine patches (i.e., label dosage recommendations will be followed) and up to 12 weeks of nicotine gum or lozenges (i.e., participants will receive a 4 week supply of NRT at the baseline visit and will be mailed NRT upon request for post-quit weeks 5-12). After 12 weeks, pharmacotherapy will be discontinued. Participants will be asked if they experienced NRT side effects each day via EMA. If/When participants report severe side effects, the app will suggest that the participant call 911. In addition, the app will automatically send an encrypted email to study staff when severe side effects are reported. Study staff will attempt to contact the participant to discuss these side effects.

*Alternative Treatments*. The consent form will explain that, rather than participating in this study, individuals may choose to enroll in the standard Tobacco Treatment Research Program (TTRP) which provides free smoking cessation medications and in-person, or phone-based smoking cessation counseling.

Human Research Participant Protection The University of Oklahoma IRB APPROVED AAHRPP
IRB NUMBER: 9860
IRB APPROVAL DATE: 06/23/2023

**5. Protections against Risk.**

*General Procedures*. Each participant will be assigned an identification number that will be utilized in place of names in all electronic and print data files. The sheet containing the links between participant names and identifiers will be kept in locked filing cabinet when not in use and will be destroyed 12 months after data collection has been completed. All print information, including informed consent and screening questionnaires, will be stored in a separate locked filing cabinet in Dr. Businelle's research offices. All electronic data (with names omitted) will be maintained on the investigators' computers, and all computers and electronic files will be encrypted and password protected. Participant information will not be released to any party outside the research team at any time.

*In-person data*. Participants will complete in-person assessments on a laptop or tablet computer using the Questionnaire Development System (QDS) program and REDCap. For QDS, participants will wear headphones and the computer will read each question aloud. Participants will be instructed that they may elect to not answer questions that make them feel uncomfortable. Participants must have a password to exit the QDS system. Thus, participants will not have access to other programs or files on the computer. In addition, all questionnaire data is automatically saved and password protected. At the end of each day, participant data will be backed up to a password protected database. Those who complete the in-person baseline via REDCap will complete the baseline questionnaires on a tablet or computer pre-loaded to the required questionnaires. The baseline surveys require a specific website link and code in order to access the questionnaire. Participants will be unable to access any other records in REDCap due to the use of this link and code. Participants will need a code to complete the final questionnaire in order to complete their baseline as well. Thus, they will not have access to any other files or programs on the tablet or computer. Alternatively, remote baseline participants will complete assessments on their personal electronic devices via a REDCap link. The REDCap surveys have been designed to be as similar to the QDS surveys as possible. Questions will not be read aloud in the REDCap version, but participants will still have the opportunity to skip questions if they choose.

To ensure that research staff are adequately trained in data collection, confidentiality, and the protection of human subjects, all project staff will complete extensive training focused on each of the following topics: 1) project rationale and objectives; 2) the informed consent process; and 3) general data collection procedures (e.g., computer data collection, privacy). They will also complete all confidentiality, conflict of interest, and HIPAA training programs as required by the University of Oklahoma Health Sciences Center.

*EMA, GPS, and CO data.* Participant responses to study questions, GPS coordinates, and results of carbon monoxide tests (i.e., Vitalograph ecolyzer and Bedfont iCO smokerlyzer) will be encrypted and stored on the study smartphone. Encrypted data will be automatically uploaded to our secure server each day. The following features are designed to ensure the security of this data: 1) the data stored on the smartphone device are in a SQLite database in a sandbox environment, where read/write operations are only available through the programming application (i.e., no file or output is readable to end users); 2) a ≥10 character password (only known to researchers) is required to authenticate the current user before data can be manually accessed; 3) the web browser application linking the investigator's computer to the database is on HTTPS protocol (SSL certificate with encryption) which will guarantee the data transfer from web browser to the backend database is well protected; and, 4) the backend database is hosted by Microsoft Azure. These steps will ensure the security of EMA, GPS, and iCO data. Software will be downloaded onto each phone so that study data can be remotely wiped at the end of the study and if a phone is lost or stolen.

Human Research Participant Protection The University of Oklahoma IRB APPROVED AAHRPP
IRB NUMBER: 9860
IRB APPROVAL DATE: 06/23/2023

Participants will use a study smartphone or their personal smartphone to complete assessments through an encrypted mobile application and all data will be automatically saved and sent to the study server. For those who receive study smartphones, the research staff will use a unique Google Play Store login to download the study app onto the phone. Passwords will only be known to research staff. At the conclusion of the participant's time in the study, participants will return the phone and all data collection through the Insight application will end. Study data will then be removed from the study phone. Participants who use their personal mobile device will receive an additional $15 per month to offset data plan costs (if at least 50% of assessments are completed and contact details are confirmed) and will use their personal Google Play Store account to download the Insight app. At the conclusion of the participant's time in the study, the study data will be removed from the participant's phone and all data collection through the Insight application will end. Researchers will give participants instructions on how to delete the app from their personal device once they complete the study.

6. **Potential Benefits.** Potential benefits to participants include the use of study phones during the intervention period (at the current time, this benefit is estimated to be unlimited voice and text messages and 2 GB high speed data for the duration of their involvement in the study [i.e., 6 months]). Participants may also directly benefit from the FREE NRT and QuitGuide or Smart-T interventions (i.e., these interventions may reduce the likelihood of smoking lapse and relapse).

It should be noted that we also have given thoughtful consideration to the ways in which the design of this research may potentially limit the generalizability and specificity of conclusions drawn from it. We opted to use the mobile Bedfont iCO to determine smoking status rather than require participants to return to the clinic for in-person follow-up visits. This approach will reduce burden for socioeconomically disadvantaged smokers who may not have reliable transportation and will ensure we have biochemical verification of quit behavior, which is central to the present stage of research development. Overall, it is expected that the potential benefits to participants in the proposed study will outweigh potential risks.

7. **Risks in relation to Benefits.** The current study involves very minimal risk to participants, and the risks of study participation are similar to that of participation in standard care (e.g., loss of confidentiality). All participants will be already seeking standard care before they are provided with any information about the research study. In addition, the knowledge gained from this study may be utilized to improve our understanding of the barriers to quitting and predictors of relapse.

**H. Data and Safety Monitoring Plan**

Procedures to minimize the risk, including of loss of confidentiality, are described in the *Protection against Risks* section. The study poses minimal risk to participants, therefore continuous monitoring and reporting of events will be undertaken by the principal investigator (Dr. Businelle) and co-investigators. Although unlikely given our experience, any unanticipated problems will be promptly reported to the IRB. The NIH will be informed of any actions taken by the IRB as a result of its continuing review. Breach of confidentiality is highly unlikely because all data will be encrypted, identified only by numeric code, and stored in locked file cabinets/online secure server. A master list of names and numbers will be kept in a separate location and is used to facilitate the collection of follow-up data. All staff will be fully trained in relevant ethical principles and procedures, particularly around confidentiality. As with any type of medication, there is the risk for unexpected side effects from the patch and gum/lozenges. Side effects will be monitored daily during daily EMA. When participants report severe side effects in the app, a secure email will be automatically sent to research staff with instructions to contact the participant to discuss. Study staff will follow-up with participants. In addition, participants will

Human Research Participant Protection The University of Oklahoma IRB APPROVED AAHRPP
IRB NUMBER: 9860
IRB APPROVAL DATE: 06/23/2023

be given contact information and instructed to call 911 and notify study staff immediately if they experience any severe side effects.



Human Research Participant Protection
The University of Oklahoma
IRB APPROVED
AAHRPP
IRB NUMBER: 9860
IRB APPROVAL DATE: 06/23/2023

# I. Literature Cited


1. Centers for Disease Control and Prevention. Annual smoking-attributable mortality, years of potential life lost, and productivity losses - United States, 1997-2001. *MMWR.* 2005;54(25):625-628.
2. Danaei G, Ding EL, Mozaffarian D, et al. The Preventable Causes of Death in the United States: Comparative Risk Assessment of Dietary, Lifestyle, and Metabolic Risk Factors. *PLoS Med.* 2009;6(4):e1000058.
3. Centers for Disease Control and Prevention. Smoking-attributable mortality, years of potential life lost, and productivity losses --- United States, 2000--2004. *MMWR.* 2008;57(45):1226-1228.
4. Jha P, Ramasundarahettige C, Landsman V, et al. 21st-century hazards of smoking and benefits of cessation in the United States. *N. Engl. J. Med.* 2013;368(4):341-350.
5. Jamal A, King BA, Neff LJ, Whitmill J, Babb SD, Graffunder CM. Current Cigarette Smoking Among Adults - United States, 2005-2015. *MMWR.* 2016(65):1205-1211.
6. U.S. Census Bureau, Quick Facts: Oklahoma. 2010-2015; http://www.census.gov/quickfacts/table/PST045215/40. Accessed April 5, 2016.
7. CDC. State Tobacco Activities Tracking and Evaluation (STATE) System. *State Highlights: Oklahoma* 2014; http://nccd.cdc.gov/STATESystem/rdPage.aspx?rdReport=OSH_STATE.Highlights. Accessed April 5, 2016.
8. U.S. Census Bureau: 2010 Census Urban and Rural Classification and Urban Area Criteria. 2010; http://www.census.gov/geo/reference/ua/urban-rural-2010.html.
9. Meit MA, Knudson A, Gilbert T, et al. The 2014 Update of the Rural-Urban Chartbook. 2014; https://ruralhealth.und.edu/projects/health-reform-policy-research-center/pdf/2014-rural-urban-chartbook-update.pdf. Accessed April 5, 2016.
10. Doescher MP, Jackson JE, Jerant A, Gary Hart L. Prevalence and Trends in Smoking: A National Rural Study. *J. Rural Health.* 2006;22(2):112-118.
11. Norris T, Vines, P. L., Hoeffel, E. M. The American Indian and Alaska Native Population: 2010. In: Commerce USDo, ed: U.S. Census Bureau; 2012.
12. Jamal A, Homa DM, O'Connor E, et al. Current cigarette smoking among adults — United States, 2005–2014. *MMWR.* 2015;64:1233-1240.
13. Pew Research Center. The Smartphone Difference. 2015; http://www.pewinternet.org/2015/04/01/us-smartphone-use-in2015/. Accessed April 3, 2015.
14. Smith A. Record shares of Americans now own smartphones, have home broadband. 2017; http://www.pewresearch.org/fact-tank/2017/01/12/evolution-of-technology/. Accessed January 15, 2017.
15. Pew Research Center. Mobile Fact Sheet. 2018; http://www.pewinternet.org/fact-sheet/mobile/. Accessed February 28, 2018.
16. Businelle MS. Advance understanding of health in homeless shelter patrons (unpublished data). University of Oklahoma Health Sciences Center; 2016.
17. Dey AK, Wac K, Ferreira D, Tassini K, Hong J-H, Ramos J. Getting closer: an empirical investigation of the proximity of user to their smart phones. Proceedings of the 13th international conference on Ubiquitous computing; 2011; Beijing, China.
18. Siahpush M, Yong HH, Borland R, Reid JL, Hammond D. Smokers with financial stress are more likely to want to quit but less likely to try or succeed: findings from the International Tobacco Control (ITC) Four Country Survey. *Addiction.* Aug 2009;104(8):1382-1390.
19. Copeland AL, Businelle MS, Stewart DW, Patterson SM, Rash CJ, Carney CE. Identifying Barriers to Entering Smoking Cessation Treatment Among Socioeconomically Disadvantaged Smokers. *Journal of Smoking Cessation.* 2010;5:164171.
20. Free C, Knight R, Robertson S, et al. Smoking cessation support delivered via mobile phone text messaging (txt2stop): a single-blind, randomised trial. *Lancet.* 2011;378(9785):49 - 55.
21. Abroms LC, Ahuja M, Kodl Y, et al. Text2Quit: Results From a Pilot Test of a Personalized, Interactive Mobile Health Smoking Cessation Program. *J Health Commun.* 2012;17(sup1):44-53.
22. Abroms LC, Lee Westmaas J, Bontemps-Jones J, Ramani R, Mellerson J. A Content Analysis of Popular Smartphone Apps for Smoking Cessation. *Am. J. Prev. Med.* 2013;45(6):732-736.
23. Smokefree.gov. QuitGuide. 2016; https://smokefree.gov/apps-quitguide.
24. Fiore MC, Jaen CR, Baker TB, et al. *Treating Tobacco Use and Dependence: 2008 Update. Clinical Practice Guideline.* Rockville, MD: Department of Health and Human Services. Public Health Service.;2008.
25. Bricker JB, Mull KE, Kientz JA, et al. Randomized, controlled pilot trial of a smartphone app for smoking cessation using acceptance and commitment therapy. *Drug Alcohol Depend.* 2014;143:87-94.
26. Shiffman S, Hufford M, Hickcox M, Paty JA, Gnys M, Kassel JD. Remember that? A comparison of real-time versus retrospective recall of smoking lapses. *J. Consult. Clin. Psychol.* Apr 1997;65(2):292-300.
27. Stone AA, Shiffman S, Schwartz JE, Broderick JE, Hufford MR. Patient non-compliance with paper diaries. *Br. Med. J.* May 18, 2002 2002;324(7347):1193-1194.
28. Holt LJ, Litt MD, Cooney NL. Prospective analysis of early lapse to drinking and smoking among individuals in concurrent alcohol and tobacco treatment. *Psychol. Addict. Behav.* Sep 2012;26(3):561-572.
29. Businelle MS, Ma P, Kendzor DE, et al. Predicting quit attempts among homeless smokers seeking cessation treatment: An ecological momentary assessment study. *Nicotine Tob Res.* 2014;16:1371-1378.
30. Lam CY, Businelle MS, Aigner CJ, et al. Individual and combined effects of multiple high-risk triggers on postcessation smoking urge and lapse. *Nicotine Tob Res.* May 2014;16(5):569-575.


Human Research Participant Protection The University of Oklahoma IRB APPROVED AAHRPP
IRB NUMBER: 9860
IRB APPROVAL DATE: 06/23/2023

31. Businelle MS, Ma P, Kendzor DE, Frank SG, Wetter DW, Vidrine DJ. Using Intensive Longitudinal Data Collected via Mobile Phone to Detect Imminent Lapse in Smokers Undergoing a Scheduled Quit Attempt. *J. Med. Internet Res.* 2016;18(10):e275.
32. Deiches JF, Baker TB, Lanza S, Piper ME. Early lapses in a cessation attempt: lapse contexts, cessation success, and predictors of early lapse. *Nicotine Tob Res.* Nov 2013;15(11):1883-1891.
33. Shiffman S, Waters AJ. Negative affect and smoking lapses: A prospective analysis. *J. Consult. Clin.Psychol.* 2004;72(2):192-201.
34. Bandiera FC, Atem F, Ma P, Businelle MS, Kendzor DE. Post-quit stress mediates the relation between social support and smoking cessation among socioeconomically disadvantaged adults. *Drug Alcohol Depend.* Jun 1 2016;163:71-76.
35. Minami H, Tran LT, McCarthy DE. Using ecological measures of smoking trigger exposure to predict smoking cessation milestones. *Psychol. Addict. Behav.* Mar 2015;29(1):122-128.
36. Shiffman S, Paty JA, Gnys M, Kassel JA, Hickcox M. First lapses to smoking: Within-subjects analysis of real-time reports. *J. Consult. Clin. Psychol.* 1996;64(2):366-379.
37. Henley SJ, Thomas CC, Sharapova SR. Vital Signs: Disparities in Tobacco-Related Cancer Incidence and Mortality - United States, 2004-2013. *MMWR.* 2016;65:1212-1218.
38. Clegg LX, Reichman ME, Miller BA, et al. Impact of socioeconomic status on cancer incidence and stage at diagnosis: selected findings from the surveillance, epidemiology, and end results: National Longitudinal Mortality Study. *Cancer Causes Control.* May 2009;20(4):417-435.
39. Browning KK, Ferketich AK, Salsberry PJ, Wewers ME. Socioeconomic disparity in provider-delivered assistance to quit smoking. *Nicotine Tob Res.* Jan 2008;10(1):55-61.
40. Hiscock R, Bauld L, Amos A, Fidler JA, Munafo M. Socioeconomic status and smoking: a review. *ANYAS.* Feb 2012;1248:107-123.
41. Piasecki TM, McCarthy DE, Fiore MC, Baker TB. Alcohol Consumption, Smoking Urge, and the Reinforcing Effects of Cigarettes: An Ecological Study. *Psychol. Addict. Behav.* 2008;22(2):230-239.
42. Reitzel LR, Kendzor DE, Nguyen N, et al. Shelter proximity and affect among homeless smokers making a quit attempt. *Am J Health Behav.* Mar 2014;38(2):161-169.
43. Kendzor DE, Shuval K, Gabriel KP, et al. Impact of a Mobile Phone Intervention to Reduce Sedentary Behavior in a Community Sample of Adults: A Quasi-Experimental Evaluation. *J. Med. Internet Res.* 2016;18(1):e19.
44. Watkins KL, Regan SD, Nguyen N, et al. Advancing cessation research by integrating EMA and geospatial methodologies: associations between tobacco retail outlets and real-time smoking urges during a quit attempt. *Nicotine Tob Res.* May 2014;16 Suppl 2:S93-101.
45. Kendzor DE, Businelle MS, Poonawalla IB, et al. Financial incentives for abstinence among socioeconomically disadvantaged individuals in smoking cessation treatment. *Am. J. Public Health.* 2015;105:1198-1205.
46. Hébert ET, Stevens, E. M., Frank, S. G., Kendzor, D. E., Wetter, D. W., Zvolensky, M. J., Buckner, J. D., Businelle, M. S. An ecological momentary intervention for smoking cessation: The associations of just-in-time, tailored messages with lapse risk factors. *Addict. Behav.* 2018;78:30-35.
47. Businelle MS, Ma P, Kendzor DE, Frank SG, Vidrine DJ, Wetter DW. An ecological momentary intervention for smoking cessation: Evaluation of feasibility and effectiveness. *J. Med. Internet Res.* 2016;18(12):e321.
48. Okuyemi KS, James, A. S., Mayo, M. S., Nollen, N., Catley, D., Choi, W. S., et al. Pathways to health: A cluster randomized trial of nicotine gum and motivational interviewing for smoking cessation in low-income housing. *Health Educ. Behav.* 2007;34(1):43-54.
49. Hahn EJ, Rayens MK, Chirila C, Riker CA, Paul TP, Warnick TA. Effectiveness of a quit and win contest with a low-income population. *Prev. Med.* 2004;39:543-550.
50. Okuyemi KS, Goldade K, Whembolua G, et al. Motivational interviewing to enhance nicotine patch treatment for smoking cessation among homelessl smokers: A randomized controlled trial. *Addiction.* 2013;108:1136-1144.
51. Christiansen BA, Reeder KM, TerBeek EG, Fiore MC, Baker TB. Motivating Low Socioeconomic Status Smokers to Accept Evidence-Based Smoking Cessation Treatment: A Brief Intervention for the Community Agency Setting. *Nicotine Tob Res.* Aug 2015;17(8):1002-1011.
52. Boland VC, Stockings EA, Mattick RP, McRobbie H, Brown J, Courtney RJ. The Methodological Quality and Effectiveness of Technology-Based Smoking Cessation Interventions for Disadvantaged Groups: a Systematic Review and MetaAnalysis. *Nicotine Tob Res.* December 29, 2016 2016.
53. Businelle MS, Cuate EL, Kesh A, Poonawalla IB, Kendzor DE. Comparing homeless smokers to economically disadvantaged domiciled smokers. *Am. J. Public Health.* 2013;103:S218-S220.
54. Businelle MS, Kendzor DE, Kesh A, et al. Small financial incentives increase smoking cessation in homeless smokers: A pilot study. *Addict. Behav.* 2014;39:717-720.
55. Businelle MS, Poonawalla IB, Kendzor DE, et al. Smoking policy change at a homeless shelter: attitudes and effects. *Addict. Behav.* Jan 2015;40:51-56.
56. Arozullah AM, Yarnold PR, Bennett CL, et al. Development and validation of a short-form, rapid estimate of adult literacy in medicine. *Med. Care.* 2007;45(11):1026-1033.
57. Department of Health and Human Services. Annual Update of the HHS Poverty Guidelines. 2016; https://www.federalregister.gov/documents/2016/01/25/2016-01450/annual-update-of-the-hhs-poverty-guidelines.


Human Research Participant Protection The University of Oklahoma IRB APPROVED AAHRPP
IRB NUMBER: 9860
IRB APPROVAL DATE: 06/23/2023

58. Mills EJ, Wu P, Lockhart I, Thorlund K, Puhan M, Ebbert JO. Comparisons of high-dose and combination nicotine replacement therapy, varenicline, and bupropion for smoking cessation: a systematic review and multiple treatment metaanalysis. *Ann. Med.* 2012;44(6):588-597.
59. North American Quit Line Cornsortium. Free and Discounted Cessation Medication. 2018; http://map.naquitline.org/reports/medication/. Accessed 1-23-18, 2018.
60. Hughes JR. An updated algorithm for choosing among smoking cessation treatments. *Journal of substance abuse treatment.* 2013;45(2):215-221.
61. Businelle MS, Kendzor DE, Cuate EL, Kesh A, Poonawalla IB. Using smart phones to collect momentary data among sheltered homeless smokers. Paper presented at: Annual Conference for the Society for Research on Nicotine and Tobacco2013; Boston, Massachusetts.
62. Shiffman S, Engberg JB, Paty JA, et al. A day at a time: Predicting smoking lapse from daily urge. *JAP.* 1997;106(1):104116.
63. Stone AA, Schwartz JE, Neale JM, et al. A comparison of coping assessed by ecological momentary assessment and retrospective recall. *JPSP.* Jun 1998;74(6):1670-1680.
64. Tobler AL, Komro KA. Contemporary options for longitudinal follow-up: Lessons learned from a cohort of urban adolescents. *Eval. Program Plann.* 2011;34(2):87-96.
65. Hughes J, Keely J, Niaura R, Ossip-Klein D, Richmond R, Swan G. Measures of abstinence in clinical trials: issues and recommendations. *Nicotine Tob Res.* 2003;5(1):13 - 25.
66. Kozlowski LT, Porter CQ, Orleans CT, Pope MA, Heatherton T. Predicting smoking cessation with self-reported measures of nicotine dependence: FTQ, FTND, and HSI. *Drug Alcohol Depend.* Feb 1994;34(3):211-216.
67. Velicer WF, Diclemente CC, Rossi JS, Prochaska JO. Relapse situations and self-efficacy: An integrative model. *Addict. Behav.* 1990;15(3):271-283.
68. Jaffee KD, Liu GC, Canty-Mitchell J, Qi RA, Austin J, Swigonski N. Race, urban community stressors, and behavioral and emotional problems of children with special health care needs. *Psychiatr. Serv.* 2005;56:63-69.
69. McCarthy WJ, Dyrness G. CPPW-L.A. Smoking Reduction Program for Transitional Shelters for the Homeless. 2012; http://publichealth.lacounty.gov/tob/pdf/bh/Bill_McCarthy_Homeles_%20Military_Communities.pdf. Accessed May 2, 2013.
70. Cohen S, Kamarck T, Mermelstein R. A gobal measure of perceived stress. *J. Health Soc. Behav.* 1983;24:385-396.
71. Cohen S, Hoberman HM. Positive events and social supports as buffers of life change stress. *J. Appl. Soc. Psychol.* 1983;13:99-125.
72. Idler EL, Benyamini Y. Self-Rated Health and Mortality: A Review of Twenty-Seven Community Studies. *J. Health Soc. Behav.* 1997;38(1):21-37.
73. Room R. Measuring alcohol consumption in the United States: Methods and rationales. In: Kozlowski LT, Annis HM, Cappell HD, et al., eds. *Research Advances in Alcohol and Drug Problems.* Vol 10. New York: Plenum Press; 1990:39-80.
74. Casey M, Hayes PS, Glynn F, et al. Patients' experiences of using a smartphone application to increase physical activity: the SMART MOVE qualitative study in primary care. *The British Journal of General Practice.* 2014;64(625):e500-e508.
75. Cottler LB, Compton WM, Ben-Abdallah A, Horne M, Claverie D. Achieving a 96.6 percent follow-up rate in a longitudinal study of drug abusers. *Drug Alcohol Depend.* 1996;41(3):209-217.
76. Shiffman S, Hickcox M, Paty JA, Gnys M, Richards T, Kassel JD. Individual differences in the context of smoking lapse episodes. *Addictive Behaviors.* 1997;22(6):797-811.
77. Wetter DW, McClure JB, Cofta-Woerpel L, et al. A randomized clinical trial of a palmtop computer-delivered treatment for smoking relapse prevention among women. *Psychol. Addict. Behav.* 2011;25:365-371.
78. Piasecki TM, Cooper ML, Wood PK, Sher KJ, Shiffman S, Heath AC. Dispositional drinking motives: Associations with appraised alcohol effects and alcohol consumption in an ecological momentary assessment investigation. *Psychol. Assess.* Jun 2014;26(2):363-369.
79. Simons JS, Dvorak RD, Batien BD, Wray TB. Event-level associations between affect, alcohol intoxication, and acute dependence symptoms: Effects of urgency, self-control, and drinking experience. *Addict. Behav.* 07/17 2010;35(12):10451053.
80. Swendsen JD, Tennen H, Carney MA, Affleck G, Willard A, Hromi A. Mood and alcohol consumption: An experience sampling test of the self-medication hypothesis. *JAP.* 2000;109(2):198-204.
81. Etter JF. Financial incentives for smoking cessation in low-income smokers: Study protocol for a randomized controlled trial. *Trials.* 2012;13.
82. Tevyaw TOL, Colby SM, Tidey JW, et al. Contingency management and motivational enhancement: A randomized clinical trial for college student smokers. *Nicotine Tob Res.* 2009;11(6):739-749.
83. Heil SH, Higgins ST, Bernstein IM, et al. Effects of voucher-based incentives on abstinence from cigarette smoking and fetal growth among pregnant women. *Addiction.* 2008;103(6):1009-1018.
84. Higgins ST, Bernstein IM, Washio Y, et al. Effects of smoking cessation with voucher-based contingency management on birth outcomes. *Addiction.* 2010;105(11):2023-2030.
85. Stoops WW, Dallery J, Fields NM, et al. An Internet-based abstinence reinforcement smoking cessation intervention in rural smokers. *Drug and alcohol dependence.* 2009;105(1-2):56-62.
86. Dunn KE, Sigmon SC, Thomas CS, Heil SH, Higgins ST. Voucher based contingent reinforcement of smoking abstinence among methadone maintained patients: A pilot study. *J. Appl. Behav. Anal.* 2008;41(4):527-538.

Human Research Participant Protection The University of Oklahoma IRB APPROVED AAHRPP
IRB NUMBER: 9860
IRB APPROVAL DATE: 06/23/2023

87. SRNT. Biochemical verification of tobacco use and cessation. *Nicotine Tob Res.* May 2002;4(2):149-159.
88. Berlin I, Radzius A, Henningfield JE, Moolchan ET. Correlates of expired air carbon monoxide: Effect of ethnicity and relationship with saliva cotinine and nicotine. *Nicotine Tob Res.* 2001;3(4):325-331.
89. Fritz M, Wallner R, Grohs U, Kemmler G, Saria A, Zernig G. Comparable sensitivities of urine cotinine and breath carbon monoxide at follow-up time points of three months or more in a smoking cessation trial. *Pharmacology.* 2010;85(4):234240.
90. Velicer WF, Prochaska JO, Rossi JS, Snow MG. Assessing outcome in smoking cessation studies. *PsyB.* 1992;111(1):2341.
91. Jarvis MJ, Tunstall-Pedoe H, Feyerabend C, Vesey C, Saloojee Y. Comparison of tests used to distinguish smokers from nonsmokers. *Am. J. Public Health.* 1987;77(11):1435-1438.
92. Bedfont. iCO Smokerlyzer. 2016; https://www.bedfont.com/shop/smokerlyzer/ico-smokerlyzer. Accessed January 9, 2017.
93. Raghunathan TE, Lepkowski JM, VanHoewyk J, Solenberger P. A multivariate technique for multiply imputing missing values using a sequence of regression models. *Survey Methodology.* 2001;27:85-95.
94. Whittaker R, McRobbie H, Bullen C, Rodgers A, Gu Y. Mobile phone-based interventions for smoking cessation. *The Cochrane database of systematic reviews.* Apr 10 2016;4:Cd006611.
95. Harkness, Audrey. (2020). The Pandemic Stress Index


Human Research Participant Protection
The University of Oklahoma
IRB APPROVED
AAHRPP

IRB NUMBER: 9860
IRB APPROVAL DATE: 06/23/2023

Human Research Participant Protection
The University of Oklahoma
IRB APPROVED
IRB NUMBER: 9860
IRB APPROVAL DATE: 06/23/2023
