# Supplementary material for: Just-in-Time Adaptive Intervention for Smoking Cessation in Low-Income Adults: A Randomized Clinical Trial
Source: JAMA Netw Open. 2025 Aug 14;8(8):e2526691. doi: 10.1001/jamanetworkopen.2025.26691 (PMC12355279; doi:10.1001/jamanetworkopen.2025.26691)
Supplement: Supplement 2. — eTable 1. Self-Reported Smoking Abstinence Rates by Treatment Group Assignment at the 26-Week Postquit Follow-Up eTable 2. On-Demand Message Access Among Smart-T Participants eTable 3. Messages viewed per Category Among Smart-T Participants eTable 4. Feature Access Among QuitGuide Participants eFigure. Study Timeline [file jamanetwopen-e2526691-s002.pdf]

Supplemental Online Content

Hébert ET, Kendzor DE, Vidrine DJ, et al. Just-in-time adaptive intervention for smoking cessation in low-income adults. *JAMA Netw Open*. 2025;8(8):e2526691. doi:10.1001/jamanetworkopen.2025.26691

eTable 1. Self-Reported Smoking Abstinence Rates by Treatment Group Assignment at the 26-Week Postquit Follow-Up

eTable 2. On-Demand Message Access Among Smart-T Participants

eTable 3. Messages viewed per Category Among Smart-T Participants

eTable 4. Feature Access Among QuitGuide Participants

eFigure. Study Timeline

This supplemental material has been provided by the authors to give readers additional information about their work.

© 2025 Hébert ET et al. *JAMA Network Open*.

eTable 1. Self-Reported Smoking Abstinence Rates by Treatment Group Assignment at the 26-Week Post-Quit Follow Up

**Abstinence Outcome**	**Total**	**Smart-T**	**QuitGuide**
7-day Self-Reported	129/352 (36.6%)	66/170 (38.8%)	63/182 (34.6%)
30-day Self-Reported	111/347 (32.0%)	59/170 (34.7%)	52/177 (29.4%)
Continuous Self-Reported	69/347 (19.9%)	37/170 (21.8%)	32/177 (18.1%)

© 2025 Hébert ET et al. *JAMA Network Open*.

eTable 2. On-Demand Message Access Among Smart-T Participants

**Message Type**	**N (Participants)**	**Mean (SD)**	**Median (IQR)**	**Range (Min–Max)**
General Quitting Advice	102	2.30 (2.52)	1.00 (1–3)	1–20
Benefits of Quitting	59	2.03 (4.00)	1.00 (1–2)	1–31
Ways to Cope with Urges	95	2.54 (3.47)	2.00 (1–3)	1–29
Ways to Cope with Stress	55	1.96 (1.55)	1.00 (1–2)	1–7
Mood Management	46	1.87 (1.65)	1.00 (1–2)	1–11
Ways to Cope with Others Smoking	38	1.74 (1.48)	1.00 (1–2)	1–8
Harms of Smoking	22	1.59 (1.33)	1.00 (1–2)	1–6
Motivation to Quit/Stay Quit	55	1.96 (2.47)	1.00 (1–2)	1–18
Nicotine Patch Information	108	1.96 (1.70)	1.00 (1–2)	1–11
Nicotine Gum/Lozenges Information	103	2.32 (2.24)	1.00 (1–3)	1–13

© 2025 Hébert ET et al. *JAMA Network Open*.

eTable 3. Messages viewed per Category Among Smart-T Participants

Message Type	N (Participants)	Mean (SD)	Median (IQR)	Range (Min-Max)
General Quitting Advice	102	43.29 (62.32)	17	1-417
Benefits of Quitting	59	27.17 (40.64)	10	1-165
Ways to Cope with Urges	95	50.17 (94.23)	17	1-601
Ways to Cope with Stress	55	11.00 (19.60)	11	1-196
Mood Management	46	14.00 (24.90)	14	1-249
Ways to Cope with Others Smoking	38	50.29 (91.71)	16	1-493
Harms of Smoking	22	46.55 (62.03)	23.5	1-247
Motivation to Quit/Stay Quit	55	39.90 (78.30)	12	1-453
Nicotine Patch Information	108	28.56 (31.10)	18	1-126
Nicotine Gum/Lozenges Information	103	31.16 (52.48)	15	1-377

© 2025 Hébert ET et al. *JAMA Network Open*.

eTable 4. Feature Access Among QuitGuide Participants

Feature	N (Participants)	Mean (SD)	Median (IQR)	Range (Min-Max)
I Was Smokefree Today	80	16.18 (31.74)	6 (2-15)	1-194
I Slipped	95	6.78 (11.52)	3 (1-7)	1-72
How to Quit	107	14.86 (54.55)	3 (1-6)	1-505
Manage My Mood	90	7.14 (13.36)	3 (1-5)	1-75
Mood Management Tips	51	3.94 (6.58)	2 (1-4)	1-43
Track Craving	97	6.48 (10.94)	3 (1-6)	1-71
Automated Tip Delivery	57	6.67 (8.29)	3 (1-6)	1-32
Journaling	70	3.74 (4.15)	2 (1-4)	1-19
Summary Statistics	81	8.44 (15.80)	4 (2-9)	1-101
Share Smoking Quit Date/Stats	24	2.63 (3.46)	1.5 (1-3)	1-17

© 2025 Hébert ET et al. *JAMA Network Open*.

eFigure. Study timeline

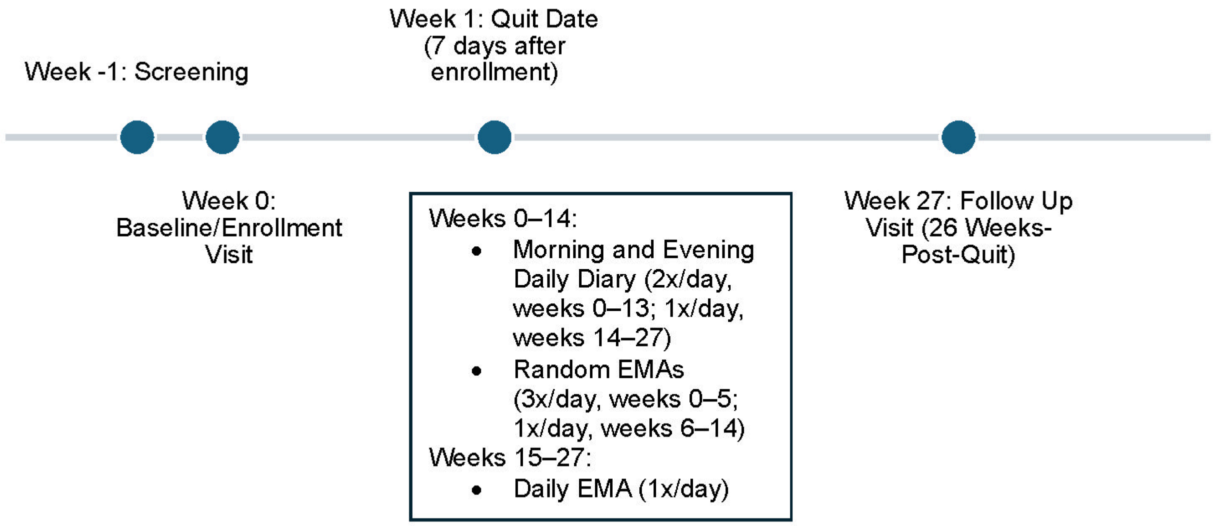

© 2025 Hébert ET et al. *JAMA Network Open*.
