# Supplementary material for: Just-in-Time Adaptive Intervention for Smoking Cessation in Low-Income Adults: A Randomized Clinical Trial
Source: JAMA Netw Open. 2025 Aug 14;8(8):e2526691. doi: 10.1001/jamanetworkopen.2025.26691 (PMC12355279; doi:10.1001/jamanetworkopen.2025.26691)
Supplement: Supplement 3. — Data Sharing Statement [file jamanetwopen-e2526691-s003.pdf]

Data Sharing Statement

Hébert. Just-in-Time Adaptive Intervention for Smoking Cessation in Low-Income Adults. *JAMA Netw Open*. Published August 14, 2025. doi:10.1001/jamanetworkopen.2025.26691

Data
Additional Information: ClinicalTrials.gov identifier: NCT03740490
Data available: Yes
Data types: Deidentified participant data, Data dictionary
How to access data: Data will be made available upon request by e-mailing michael-businelle@ouhsc.edu.
When available: With publication

Supporting Documents
Document types: None

Additional Information
Who can access the data: Researchers and others with university approved requests.
Types of analyses: All reasonable requests will be entertained.
Mechanisms of data availability: We will follow all university requirements for data sharing with individuals who have reasonable data requests.
